# Comprehensive immune profiling reveals that *Orbivirus* infection activates immune checkpoints during acute T cell immunosuppression

**DOI:** 10.3389/fimmu.2023.1255803

**Published:** 2023-10-18

**Authors:** Andrés Louloudes-Lázaro, José M. Rojas, Isabel García-García, Daniel Rodríguez-Martín, Esther Morel, Verónica Martín, Noemí Sevilla

**Affiliations:** ^1^ Centro de Investigación en Sanidad Animal, Instituto Nacional de Investigación y Tecnología Agraria y Alimentaria, Consejo Superior de Investigaciones Científicas (CISA-INIA-CSIC), Madrid, Spain; ^2^ Departamento de Genética, Fisiología y Microbiología, Unidad de Genética, Facultad de Ciencias Biológicas, Universidad Complutense de Madrid (UCM), Madrid, Spain

**Keywords:** *Orbivirus*, T cells, PD-1/PD-L1 checkpoint, transcriptomic, monocytes, B cells

## Abstract

Bluetongue virus (BTV) is an arbovirus transmitted by the bite of infected *Culicoides* midges that affects domestic and wild ruminants producing great economic losses. The infection induces an IFN response, followed by an adaptive immune response that is essential in disease clearance. BTV can nonetheless impair IFN and humoral responses. The main goal of this study was to gain a more detailed understanding of BTV pathogenesis and its effects on immune cell populations. To this end, we combined flow cytometry and transcriptomic analyses of several immune cells at different times post-infection (pi). Four sheep were infected with BTV serotype 8 and blood samples collected at days 0, 3, 7 and 15pi to perform transcriptomic analysis of B-cell marker^+^, CD4^+^, CD8^+^, and CD14^+^ sorted peripheral mononuclear cells. The maximum number of differentially expressed genes occurred at day 7pi, which coincided with the peak of infection. KEGG pathway enrichment analysis indicated that genes belonging to virus sensing and immune response initiation pathways were enriched at day 3 and 7 pi in all 4 cell population analyzed. Transcriptomic analysis also showed that at day 7pi T cell exhaustion pathway was enriched in CD4^+^ cells, while CD8^+^ cells downregulated immune response initiation pathways. T cell functional studies demonstrated that BTV produced an acute inhibition of CD4^+^ and CD8^+^ T cell activation at the peak of replication. This coincided with PD-L1 upregulation on the surface of CD4^+^ and CD8^+^ T cells as well as monocytes. Taken together, these data indicate that BTV could exploit the PD1/PD-L1 immune checkpoint to impair T cell responses. These findings identify several mechanisms in the interaction between host and BTV, which could help develop better tools to combat the disease.

## 1 Introduction

Bluetongue (BT) is an infectious disease transmitted by *Culicoides* midges caused by Bluetongue virus (BTV), which is the prototype member of the *Orbivirus* genus within the *Sedoreoviridae* family (1). BTV genome consists of 10 double-stranded RNA segments encoding 7 structural proteins (VP1-VP7) and at least 4 non-structural proteins (NS1-NS4) (2, 3). This segmented genome favors the appearance of new serotypes due to the possibility of segment reassortment during co-infection with different serotypes (4, 5). So far up to 29 serotypes have been described (6), although the coexistence of multiple serotypes in the same territory leads to the ongoing emergence of new strains (7). Similarly, the use of live attenuated vaccines could contribute to the appearance of new serotypes, as vaccine strains can reassort with wild type circulating virus (8, 9).

BTV is currently spread through Asia, the Middle East, Australia, South and Central America and Southern Europe, making it a disease of compulsory notification to the WOAH (World Organization for Animal Health) (10). Sheep, goats, cattle and wild ruminants are the main hosts of the disease, which causes high rates of mortality in naïve herds, abortions, weight loss and decrease in fertility and milk production that result in great economic losses for the livestock industry (11–13).

Following the bite of an infected midge, BTV initially replicates in endothelial cells before infecting lymphocytes and cells of the monocytic lineage (14–17). The virus is then transported through the lymph, colonizing the draining lymph nodes and spreading to peripheral tissues (18).BTV is also capable of “hiding” in erythrocytes, which promotes the spread to other tissues (19). BTV has the particularity to produce prolonged infections in ruminants, which probably favors the transmission of the virus back to the *Culicoides* spp. vector. The host can be infectious for biting midges for at least 21 days; infectious virus can be isolated from blood for up to 49 days; and in some cases, viral RNA can be detected in blood for more than 200 days (20). The prolonged viremia indicates that BTV has mechanisms that allow it to evade immunity to extend its circulation in the host.

BTV can produce leucopenia during the early stages of experimental infections (21–24) and this could be due to virus-related apoptosis of peripheral blood mononuclear cells (23). The infection not only affects leucocytes in the periphery but also in the lymph nodes (25). Indeed, BTV is known to be transported to the draining lymph nodes by conventional DC (26). Once in the lymph node, BTV disrupts follicular DC activity, which results in delayed antibody responses (27). BTV effects on cellular immunity are less well characterized, but early evidence suggested that BTV could produce transient T cell immunosuppression (22). It is therefore critical to understand the interaction of BTV with the host immune system to further our knowledge of BTV infections.

Humoral and cell-mediated immunity play a critical role to overcome the disease. Although neutralizing antibodies can confer long-lasting protection against reinfection with a homologous BTV serotype (28), cellular immunity can confer protection against homologous and heterologous BTV infection in the absence of neutralizing antibodies (29–32). CD4^+^ and CD8^+^ T-lymphocytes, which are activated upon antigen presentation, govern cell-mediated immune responses to BTV that typically result in CD8^+^ T-cell expansion two weeks after BTV infection (21, 24).

Upon BTV infection, dsRNA is detected by pattern recognition receptors (PRRs), in particular toll-like receptors and retinoic acid inducible gene (RIG-1)-like family receptors (33), triggering the production of interferon and other proinflammatory cytokines to activate an antiviral response to combat the infection. Despite the studies on transcriptome analysis of BTV infected peripheral blood mononuclear cells (PBMCs) confirming that part of the differentially expressed genes (DEGs) during infection are involved in PRR activation and induction of cytokine signaling (34), little information is known about the transcriptomic characterization of the immune response against BTV. To date, there have been no *in vivo* reports of transcriptome profiling of PBMC subpopulations in BTV-infected sheep. We have therefore carried out in the present study a transcriptomic analysis of B, CD14^+^, CD4^+^ and CD8^+^ cells from four sheep infected with BTV-8. In addition, transcriptomic data have been contrasted with functional analysis by flow cytometry and interferon production assays. The present study furthers our understanding of BTV immunomodulatory activity on the host immune cells.

## 2 Materials and methods

### 2.1 Ethical statement

Animal experiments were carried out in a biosafety level 3 facility at the Centro de Investigación en Sanidad Animal (CISA). Animal suffering was minimized according to the recommendations in the guidelines of the Code for Methods and Welfare Considerations in Behavioral Research with Animals (Directive 86/609EC; RD1201/2005). Experiments were approved by the Committee on the Ethics of Animal Experiments of the Spanish Instituto Nacional de Investigación y Tecnología Agraria y Alimentaria (INIA) and the National Animal Welfare Committee (PROEX 032/19).

### 2.2 Animal experiments

Four sheep from the “Churra” breed between one and a half and two-years-old were housed in the same room in appropriate containment facilities with food and water ad libitum, controlled temperature and light/dark cycles. Animals were monitored daily during an acclimatization period of two weeks prior to the beginning of the experiment.

Sheep were inoculated with 2 ml (1×10^7^ PFU/ml) of BTV-8 (NET2006/04). One milliliter was administered intravenously, and the other milliliter was administered intradermally divided in multiple inoculations in the axillary and inguinal areas. Blood samples were collected at day 0 prior to infection and at days 3, 7, 10, 15 and 17 post-infection. Animals were sacrificed at day 17 post-infection.

Rectal body temperature and clinical signs based on food intake, depression, facial and foot lesions, respiratory signs and fever were recorded daily and scored as described in (35). No animals died during the experiment nor did they reach scores high enough to consider euthanasia. At the end of the experiment animals were euthanized by intravenous administration of ketamine 100 mg/ml (2.2 mg/kg), atropine 1 mg/ml (0.05 mg/ml), and xylazine 20 mg/ml (0.15 mg/kg) for sedation, followed by a lethal dose of pentobarbital sodium (200 mg/ml) 1 ml/1.5 kg.

### 2.3 Peripheral blood mononuclear cells isolation

Peripheral blood mononuclear cells (PBMCs) were obtained from 100 ml of total blood collected in EDTA from jugular vein at day 0 prior to infection and days 3, 7 and 15 post-BTV infection. The samples were purified using Ficoll cushion (GE Healthcare) purification method as described in (36). PBMCs were frozen as described in (36). When necessary, PBMCs were cultured in PBMC culture medium (RPMI supplemented with 10% FBS (Sigma), 4 mM L-glutamine, 10 mM HEPES, 1% 100X non-essential aminoacids, 1 mM sodium pyruvate, 100U/mL penicillin/100 μg/mL streptomycin and 50nM β-mercaptoethanol (all from Invitrogen)).

### 2.4 Flow cytometry analysis and cell sorting of PBMCs

PBMCs subpopulations were stained with the following antibodies: anti-ovine CD4 (clone 44.38), CD8 (clone 38.65), anti-human CD14 (clone TÜK4) and CD16 (clone KD1) (all from Bio-Rad). Regarding staining of B cells, we have used the anti-bovine B cell marker (clone BAQ44A) (Kingfisher Biotech). As mentioned in the commercial datasheet the CD equivalent for the anti-B cell marker antibody is unknown. We found that the B cell marker antibody labeled a broader population of B cells than CD21, since B cell marker^+^ CD21^+^ and B cell marker^+^ CD21^-^ cells were present in sheep PBMC. We therefore chose the anti-B cell marker for sorting as it allowed us to obtain a broader spectrum of B cells for transcriptomic analysis. Briefly, cells were washed twice with PBS and stained with LIVE/DEAD™ Fixable Near-IR Dead Cell Stain Kit (Invitrogen) for 20 minutes on ice. Then cells were washed twice with PBS + 2% FBS + 0.02% sodium azide before staining for 20 minutes on ice with the appropriate fluorochrome-conjugated antibodies. Unconjugated B-cell marker antibody was conjugated with Lightning-Link® Rapid Alexa Fluor 647 Antibody Labeling Kit (Novus Biologicals). Samples were either run on a FACSCelesta™ SORP cytometer (BD Biosciences) for flow cytometry analysis or on a FACSAria™ III sorter (BD Biosciences) to sort B cell marker^+^, CD14^+^, CD4^+^ and CD8^+^ populations. Sorting was performed on freshly isolated PBMC, while phenotypic characterization of CD14^+^ cell was performed on defrosted PBMC. Sorting purity of >95% was achieved for all cell populations. Gating strategy for cell sorting is presented in **Supplementary Figure 1**. We have found in previous experiments that CD8^high^ cells are negative for NK cell markers CD16 and CD335, thus NK cells contamination is likely minimal in this fraction. In accordance with other studies (37), we also found that γδ T cells are typically CD4^-^ and CD8^-^ in the periphery in sheep, therefore CD4^+^ and CD8^+^ fractions mainly consisted of conventional αβ T cells, although a small contamination with γδ T cells cannot be excluded. The number of sorted cells are shown in **Supplementary Table 1**. Appropriate isotype and fluorescence minus one channel (FMO) controls were included in all staining.

For PD-L1 staining, rabbit anti-bovine PD-L1 polyclonal antibody (reference KP1917B-100 from Kingfisher Biotech) was used. Thawed PBMCs were stained with anti-PD-L1 antibody and anti-Rabbit Alexa Fluor 647 secondary antibody (ThermoFisher) prior to CD4, CD8 and CD14 staining as described above. Dead cells were labeled with LIVE/DEAD™ Fixable Near-IR Dead Cell Stain Kit (Invitrogen). Appropriate isotype and FMO controls were performed for PD-L1 gate setting (**Supplementary Figure 2**). Sample acquisition was performed on a FACSCelesta™ SORP cytometer (BD Biosciences). FlowJo software (TreeStar Inc.) was used for flow cytometry analysis.

### 2.5 Intracellular cytokine staining of ovine IFN-γ and flow cytometry proliferation assays

ICS assays were carried out according to (36, 38). Briefly, defrosted PBMCs were incubated with concanavalin-A (ConA) (1.25 μg/ml) or with phorbol 12-myristate 13-acetate (PMA) (50 ng/ml) plus ionomycin (1 μg/ml) in presence of 10 μg/ml brefeldin-A (Biolegend) for 4 h at 37°C, 5% CO_2_. PBMCs were then washed twice with PBS and stained with LIVE/DEAD™ Fixable Near-IR Dead Cell Stain Kit (Invitrogen) for 20 minutes on ice before staining with anti-ovine CD4-FITC and CD8-PE antibodies (both from BioRad). Cells were then fixed and permeabilized following the instructions of the Cytofix/CytoPerm kit (BD Biosciences) and stained with anti-bovine IFN-γ-A647 (clone CC302; Bio-Rad) antibody. Appropriate isotype and FMO controls were included. Gating strategy is detailed in **Supplementary Figure 3**.

Proliferation assays were performed as described in (39). Briefly, thawed PBMCs were stained with CellTrace Violet according to the manufacturer’s protocol and stimulated with ConA (1.25 μg/ml) or staphylococcus enterotoxin B (SEB) (1 μg/ml). After 96h, PBMCs were stained with LIVE/DEAD™ Fixable Near-IR Dead Cell Stain Kit (Invitrogen) for 20 minutes on ice before staining with anti-ovine CD4-AlexaFluor647 and CD8-PE antibodies (both from BioRad). Gating strategy of proliferating cells is detailed in **Supplementary Figure 4**. Sample acquisition was performed on a FACSCelesta™ SORP cytometer (BD Biosciences) and analysis performed with the FlowJo software (TreeStar Inc.).

### 2.6 IFN-γ ELISpot assays

Ovine IFN-γ ELISpot were carried out as described in (36) on defrosted PBMC using MSIPS4510 plates (Milipore). Membranes were incubated with anti-ovine IFN-γ antibody (MT17.1, Mabtech) and blocked with PBMC culture medium after extensive washing. Cells were plated at a density of 2–3 × 10^5^ cells per well and incubated with BEI inactivated BTV-8 (i-BTV), recombinant BTV-VP7 protein, PBMC culture medium as a negative control or ConA (1.25 μg/ml) as a positive control. Membranes were then incubated with biotin-labelled anti-ovine IFN-γ antibody (MT307-biotin, Mabtech) and developed with streptavidin conjugated to alkaline phosphatase (ExtrAvidin-AP, Sigma). Finally, membranes were revealed using SigmaFAST BCIP/NBT (Sigma). ELISpot plate counts were determined using an AID iSpot Reader System (Autoimmun Diagnostika GMBH). ELISpot assays were considered valid when counts in negative control wells were below 25 spots.

### 2.7 Viral RNA extraction and qPCR quantification

RNA was extracted from blood samples using IndiSpin Pathogen Kit (Indical Bioscience) following the manufacturer´s guidelines. Reverse transcription quantitative PCR (RT-qPCR) was carried out using Luna® Universal One-Step RT-qPCR Kit (New England Biolabs). Primers Forward 5`-GTTGAATTGGCAAAGGAGGCAATG-´3 and Reverse 5´-GGGATGATGGATGAGGCCGTG-3´ were used to amplify 165pb fragments of segment 5 of BTV. MX3005P thermocycler (Agilent) was used for viral RNA quantification.

### 2.8 RNA sequencing and raw data processing

RNA from freshly isolated PBMC that were subsequently sorted for B cell marker^+^, CD14^+^, CD4^+^ and CD8^+^ cells at days 0, 3, 7 and 15 post-infection was isolated using RNeasy Mini Kit (Qiagen) following the manufacturer´s guidelines. RNA samples were treated with RNase-Free DNase (Qiagen) to remove genomic DNA. RNA quality and quantity was measured using an Agilent Technologies 2100 Bioanalyzer. 59 samples scored a RNA integrity number (RIN) of 7.0 or higher and were used for RNA sequencing. SMARTer Ultra Low RNA Kit was used for RNA library preparations, which were then sequenced on Illumina NovaSeq6000 sequencing system (150PE, 20 M reads/sample).

Quality of raw reads was checked using FastQC v0.11.9 (40). Adapter sequences were trimmed and low-quality reads were removed using fastp v0.20.1 (41), retaining reads with a Phred score higher than 15 and with more than 36 bases.

### 2.9 Differential gene expression analysis and KEGG pathway analysis

The remaining high-quality reads were mapped to the *Ovis aries* reference genome using subread-align (42) and gene counts were obtained using featureCounts (43), both included in the Subread v2.0.3 package. Genes with low counts (below 15 reads per group) were removed, counts were normalized and differentially expressed genes (DEGs) were identified using edgeR v3.32.1 (44). B cell marker^+^, CD4^+^, CD8^+^ and CD14^+^ sorted cell samples were individually analyzed, comparing days 3, 7 and 15 post-infection to day 0 (prior to infection). An FDR correction was applied and genes with a q-value<0.05 were considered as DEGs. Relevant DEGs were selected based on the gene ontology (GO) terms associated to each gene. GO terms were obtained using biomaRt v2.46.3 (45).

An over-representation analysis (ORA) was performed to identify pathways enriched in DEGs, using clusterProfiler v3.18.1 (46). The *Ovis aries* specific version of the KEGG (Kyoto Encyclopedia of Genes and Genomes) database was used. An FDR correction was applied and those pathways with a q-value<0.05 were considered significantly enriched in DEGs.

### 2.10 Statistical analysis

Data handling analyses were performed using Prism 6.0 (GraphPad Software Inc., San Diego, CA, USA). Statistical tests used for analysis are described in the figure legend.

## 3 Results

### 3.1 BTV infection induces a decline in CD8^+^-T cells during acute infection and an increase in CD14^+^ CD16^+^ monocytes

**Figure 1:**
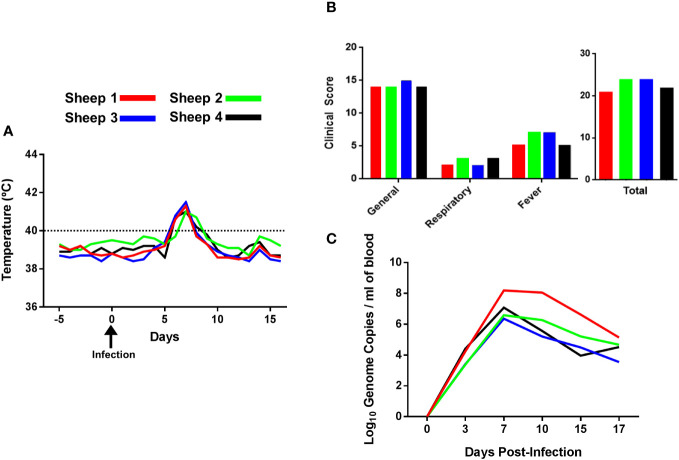
Rectal body temperature, clinical signs and viremia of sheep infected with BTV-8. **(A)** The rectal body temperature of four sheep was recorded daily during 5 days prior to infection and 16 days post-infection. Body temperatures above 40°C (denoted in the graph with a dotted line) were considered fever. **(B)** Clinical signs of BTV-infected sheep. Animals were scored daily after infection using a clinical index based on general signs, respiratory signs, fever, need for veterinary intervention or death as described in Materials and Methods. The total scores for each individual animal are given as the sum of the value for each sign observed during 16 days after BTV infection. **(C)** BTV RNA extracted from blood samples detected by RT-qPCR and expressed as log_10_ genome copies per milliliter of blood.

Four sheep were infected with a virulent strain of BTV-8 and rectal body temperature and clinical signs were monitored on a daily basis up to day 17 post-infection (pi) (**Figure 1A**; **Supplementary Figure 5**). All animals showed a temperature above 40°C at days 6, 7 and 8 pi, which started to decline at day 9 pi. Furthermore, the clinical score showed an average of 22.75 ± 1.75, with all sheep developing moderate to severe clinical signs between day 6 and 8 pi (**Figure 1B**). RT-qPCR of BTV RNA was carried out using blood samples collected prior to infection (day 0) and at days 3, 7, 10, 15 and 17 post-infection to determine viremia (**Figure 1C**). The maximum number of log_10_ genome copies of viral RNA per milliliter of blood of all four sheep was detected at day 7 post infection. Although viremia started to decrease at day 10, viral RNA was still detectable at day 17 post-infection. We did not detect significant changes in total PBMC counts post-infection in this experiment (**Supplementary Figure 6A**), thus this experimental infection did not induce significant leucopenia.

**Figure 2:**
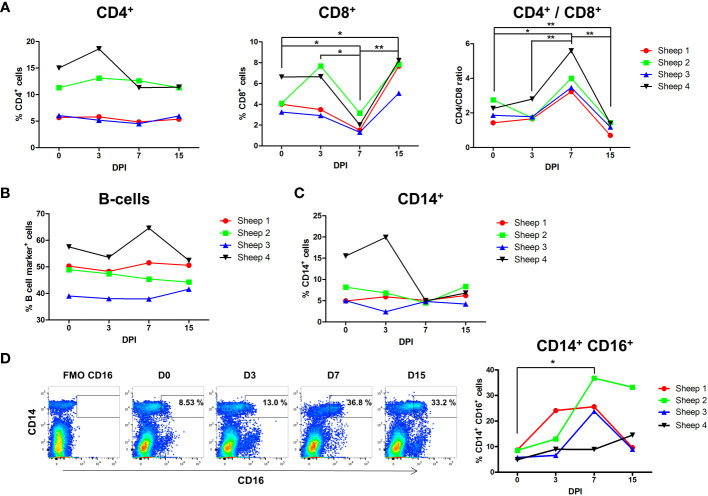
Flow cytometry analysis of leukocyte populations during BTV infection. Four sheep were infected with BTV-8 and percentage of cell variation of CD4^+^, CD8^+^, B and CD14^+^ cells was studied at day 0 (prior to infection) and at days 3, 7 and 15 post-infection (DPI). **(A)** Percentage of CD4^+^ and CD8^+^ cells in PBMCs and ratio of CD4^+^/CD8^+^ cells. Percentage of **(B)** B-cells and **(C)** CD14^+^ cells. **(D)** Representative dot-plots of CD14 and CD16 staining within the monocyte gate at the different timepoints assessed are shown. Fluorescence minus one (FMO) channel for CD16 was used to gate CD16^+^ monocytes. Percentage of CD16^+^ cells in the CD14^+^ gate for all 4 infected sheep is plotted. *p< 0.05; **p< 0.01; One-way ANOVA with Fisher’s LSD post-test.

To assess the dynamic fluctuations of immune cells during acute infection with BTV-8, CD14^+^, CD4^+^ and CD8^+^ and B cells were analyzed by flow cytometry at days 0, 3, 7, and 15pi. While CD4^+^ cells percentage did not show significant differences throughout the infection (**Figure 2A**), CD8^+^ cell percentage significantly decreased by day 7pi and subsequently significantly increased by day 15pi above levels seen prior to the infection. These findings were confirmed when CD4^+^ and CD8^+^ cell percentage were normalized to PBMC counts, which established CD8^+^ cell numbers decreased at day 7pi and subsequently expanded at day 15pi (**Supplementary Figures 6B, C**). The CD4^+^/CD8^+^ ratio was maintained constant up to day 7pi, at which point a significant CD4^+^/CD8^+^ ratio increase was observed that then significantly declined at day 15pi (**Figure 2A**). It is unlikely that the circulating CD8^+^ T cells contraction we detect at day 7pi is due to the recruitment of T cells at the site of infection, since previous reports indicated the CD8^+^ T cells expanded in the proximal lymph day by day 10 during primary responses to BTV (25). This data rather suggest a migration of CD8^+^ cytotoxic T lymphocytes (CTL) to secondary lymphoid organs to mount an immune response, and therefore a reduction of their number in the periphery, or a depletion of CD8^+^ T cells. It is however noteworthy that CD4^+^ T cells did not expand at any time post-infection, supporting the hypothesis that CD8^+^ T cell depletion occurs during BTV acute infection. Similarly to CD4^+^ T cells, the percentage and number of B cells (**Figure 2B**, **Supplementary Figure 6D**) did not change during acute infection, further indicating that depletion of circulating CD8^+^ T cells could be taking place.

Monocytes are one of the main targets of BTV infection in ovine and bovine PBMCs (17, 47). No significant changes in cell percentage or number of monocytes (CD14^+^) were observed during BTV infection (**Figure 2C**, **Supplementary Figure 6E**). However, flow cytometry analyses of CD14^+^CD16^+^ monocytes showed that CD14^+^ CD16^+^ cell percentage significantly increased at day 7pi (**Figure 2D**). Based on bovine data, this monocyte subset could putatively represent in sheep a subpopulation of intermediate/non-classical monocytes (48, 49). Alternatively, this subset could also contain a classical monocyte population activated as a result of the infection (49). Overall, it appears that the CD14^+^ CD16^+^ monocyte subset could be relevant in the response to BTV infection.

### 3.2 Transcriptome profiling of B, CD14^+^, CD4^+^ and CD8^+^ cells

Our previous findings showed a differential expansion of CD4^+^, CD8^+^, B-cells and CD14^+^CD16^+^ monocytes during BTV infection. To gain a deeper understanding of those genes and/or pathways directly or indirectly linked with BTV infection, B cell marker^+^, CD14^+^, CD4^+^ and CD8^+^ cells were sorted from freshly isolated PBMCs from blood samples collected prior to infection (day 0) and at days 3, 7 and 15 pi in these four BTV-infected sheep. RNA was isolated from these subsets to profile the transcriptome of each cell population through the course of the infection.

#### 3.2.1 Differentially expressed genes

**Figure 3:**
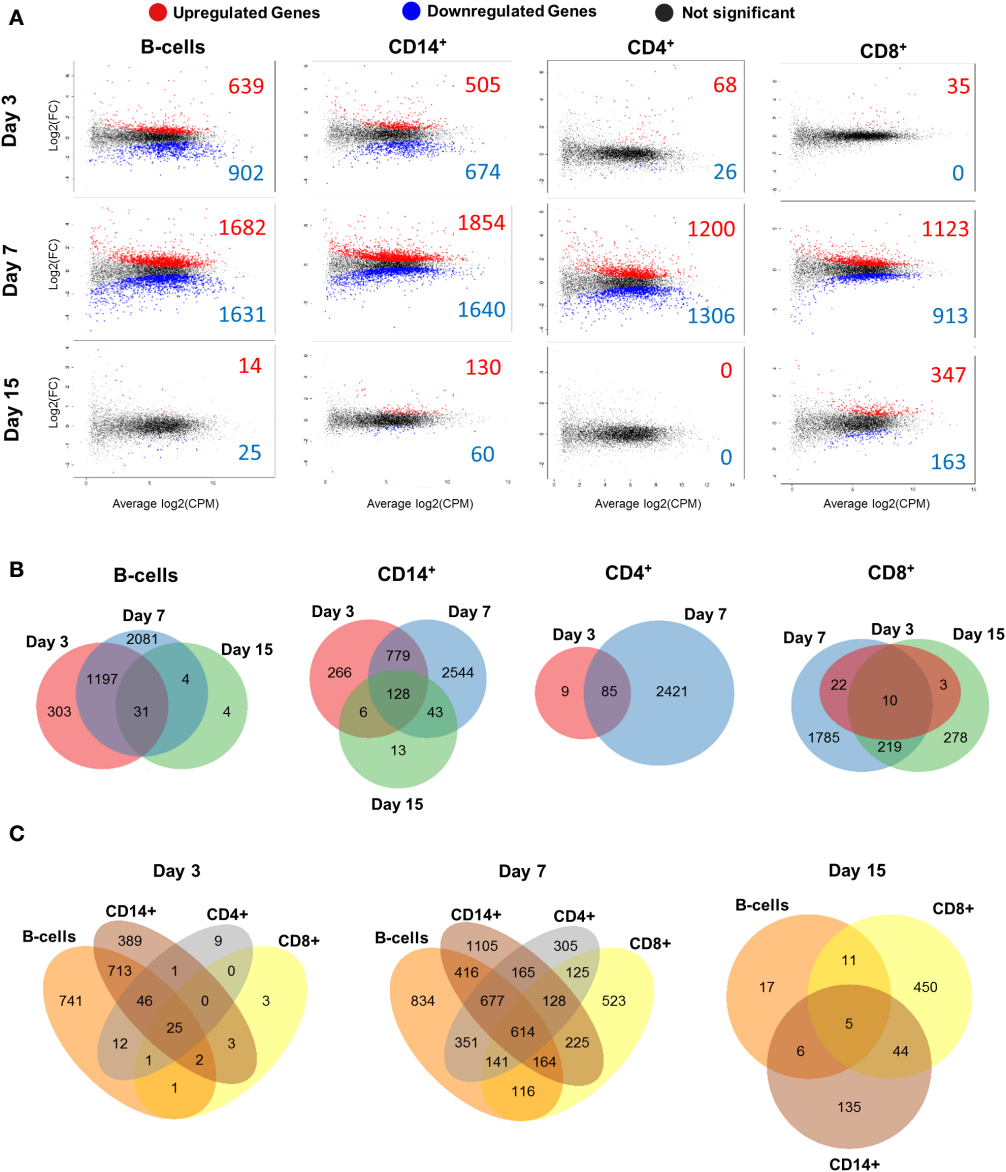
Differentially expressed genes (DEGs) of B, CD14^+^, CD4^+^ and CD8^+^ cell populations at different times during BTV infection. **(A)** Mean-Difference (MD) plots showing gene expression of sorted B, CD14^+^, CD4^+^ and CD8^+^ cells at days 3, 7 and 15 post-infection with BTV. Gene expression is given as the log2 fold change **(FC)** versus the average log2 count per million (CPM). Significantly upregulated genes are represented as red dots and significantly downregulated genes are represented as blue dots, whilst genes with no significant changes are represented as black dots. **(B)** Venn diagrams indicating the number of DEGs for B, CD14^+^, CD4^+^ and CD8^+^ cell population at days 3, 7 and 15 post-infection. Unique/common DEG numbers between timepoints are indicated on the diagrams. **(C)** Venn diagrams with number of unique/common DEGs between cell populations at days 3, 7 and 15 post-infection. Only samples with RIN>7.0 were processed for RNAseq (59 out of 64 possible samples).

The total number of differentially expressed genes (DEGs) in B cells at day 3 was 1541 (639 upregulated and 902 downregulated), 3313 at day 7 (1682 upregulated and 1631 downregulated) and 39 at day 15 (14 upregulated and 25 downregulated) (**Figure 3A**; **Supplementary File 1**). CD14^+^ cells had 1179 DEGs at day 3 (505 upregulated and 674 downregulated), 3494 at day 7 (1854 upregulated and 1640 downregulated) and 190 at day 15 (130 upregulated and 60 downregulated). CD4^+^ cells showed 94 DEGs at day 3 (68 upregulated and 26 downregulated), 2506 at day 7 (1200 upregulated and 1306 downregulated) and no DEGs at day 15 were detected. CD8^+^ cells presented 35 DEGs at day 3 (all upregulated), 2036 at day 7 (1123 upregulated and 913 downregulated) and 510 at day 15 (347 upregulated and 163 downregulated) (**Figure 3A**; **Supplementary File 1**). B cells and CD14^+^ cells had higher number of DEGs at day 3 compared to CD4^+^ and CD8^+^ cells. All cell populations showed maximum numbers of DEGs at day 7, which decreased at day 15. CD8^+^ cells showed the highest number of DEGs at day 15 of all 4 populations studied.

Venn diagrams were generated to assess the DEG overlap between timepoints for each cell population (**Figure 3B**). The highest number of common DEGs between days 3 and 7 was observed in B and CD14^+^ cells, whilst CD4^+^ and CD8^+^ T-cells presented fewer common genes between these days. CD8^+^ cells shared the highest number of common DEGs between days 7 and 15 of all the cell population analyzed. The transcriptomic profile of CD8^+^ cells was nonetheless quite different between day 7pi and 15pi since approximately only 10% of day 7 DEGs (229 out of 2036 DEGs) were still expressed at day 15. Additionally, other Venn diagrams were generated to study the DEGs shared between cell populations at the different times post-infection (**Figure 3C**). At days 3pi and 7pi, B and CD14^+^ cells shared the highest number of common DEGs, while CD4^+^ and CD8^+^ cells shared the lowest. Interestingly, at day 7pi, 614 DEGs are common between all cell populations analyzed, and most DEGs expression overlaps more than one cell population. This could indicate that an immune response is taking place in all four cell populations. At day 15, the highest number of DEGs was shared between CD14^+^ and CD8^+^ cells, but transcriptomic profiles were quite distinct between B-cell, CD14^+^ and CD8^+^ cell populations as most DEG expression was unique to each cell population. Overall, transcriptomic analysis of B, CD14^+^, CD4^+^ and CD8^+^ cells indicates that BTV infection starts altering the activity of these immune cells early in the infection (by day 3pi). Changes in transcriptomic profile peaked at a day 7pi and subsequently appear to normalize by day 15.

#### 3.2.2 Kyoto encyclopedia of genes and genomes pathways enrichment analysis

**Figure 4:**
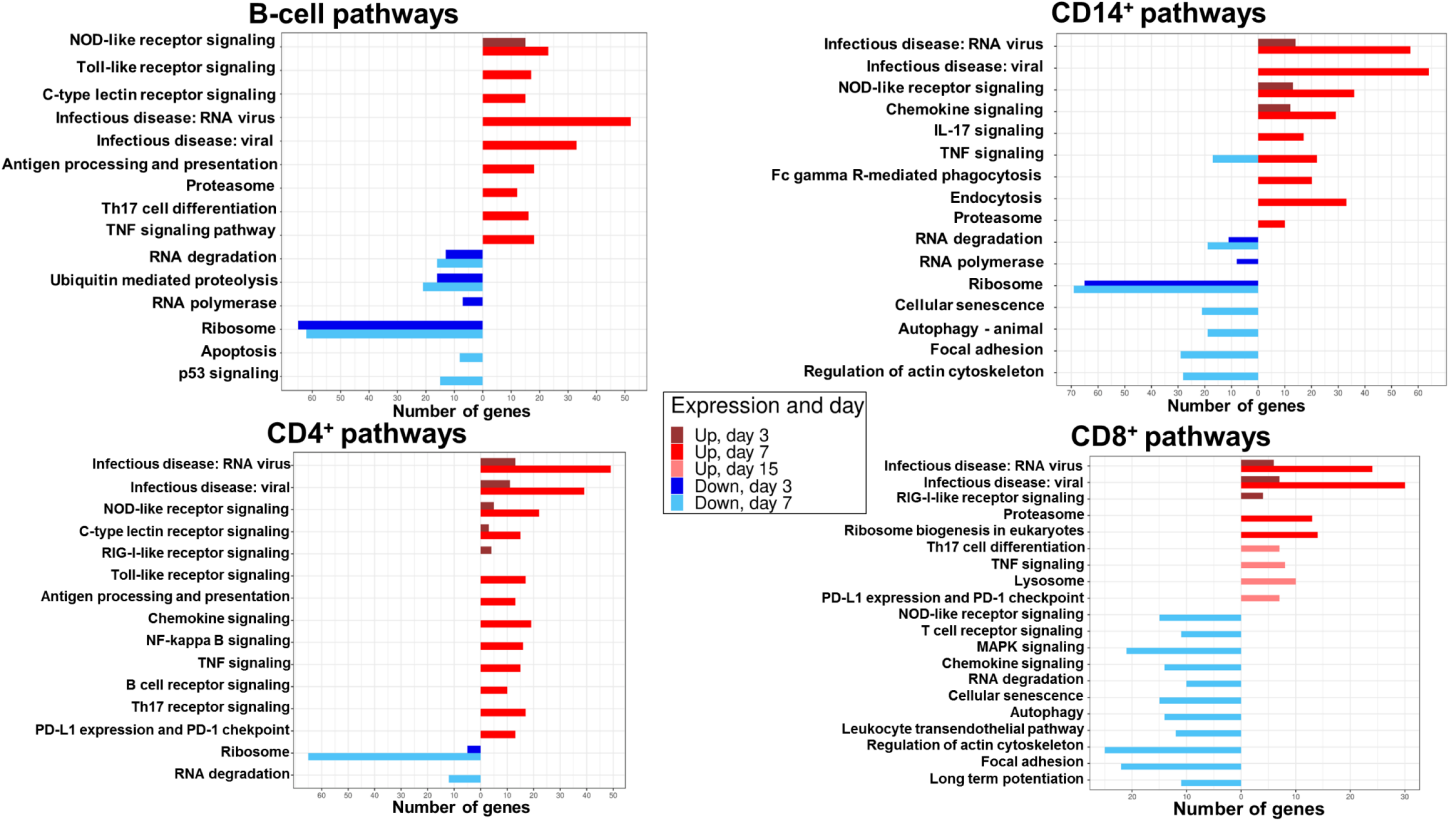
DEG enrichment analysis by ORA-KEGG in B, CD14^+^, CD4^+^ and CD8^+^ cell populations at different times post-BTV infection. The number of genes upregulated (red) or downregulated (blue) in statistically significant signaling pathways identified by ORA-KEGG is plotted in sorted B, CD14^+^, CD4^+^, and CD8^+^ cells at days 3, 7, and 15 pi. Only samples with RIN>7.0 were processed for RNAseq (59 out of 64 possible samples).

To better understand the biological pathways modulated by the infection in B, CD14^+^, CD4^+^ and CD8^+^ cells, we next performed an over-representation analysis (ORA) using the Kyoto Encyclopedia of Genes and Genomes (KEGG) (**Figure 4**).

##### 3.2.2.1 B-cells

At day 3, genes belonging to pattern recognition receptor (PRR) signaling (NOD-like receptor signaling) pathway were upregulated, while genes involved in RNA degradation, ubiquitin mediated proteolysis, RNA polymerase, and ribosome pathways were downregulated. By day 7, a further increased expression in genes related to PRR signaling was detected (NOD-like receptor signaling, toll-like receptor signaling and C-type lectin receptor signaling pathways). Concomitantly, genes related to viral sensing, antigen processing and presentation, Th17 cell differentiation and cytokine signaling (TNF signaling) were upregulated. Genes involved in RNA degradation, ubiquitin mediated proteolysis and ribosome remained downregulated at day 7. Apoptosis (p53 signaling) pathways were also downregulated at this timepoint in B cells. No pathway enrichment was detected at day 15 (**Figure 4**).

ORA-KEGG analysis indicates that B-cell DEGs were mainly involved in the sensing of viral infection and initiation of an immune response against the infection, and that these pathways are mostly upregulated at day 7 post-infection.

##### 3.2.2.2 CD14^+^ cells

Upregulated DEGs detected in CD14^+^ cells at day 3 were involved in virus sensing (infectious disease: RNA virus and infectious disease: viral), PRR signaling (NOD-like receptor signaling), and chemokine signaling (**Figure 4**). Similarly to B cells, downregulated genes at day 3 belonged to pathways related to RNA degradation, RNA polymerase and ribosome. At day 7, a further increase in upregulated DEGs in viral sensing, PRR signaling (NOD-like receptor), and cytokine signaling (chemokine, IL-17 and TNF signaling) pathways was detected. Furthermore, upregulated DEGs in phagocytosis, endocytosis, and antigen processing (proteasome) pathways were also identified. Downregulated genes at day 7 were involved in RNA degradation, ribosome, cellular senescence, autophagy, focal adhesion, and regulation of actin cytoskeleton pathways. No pathway enrichment was detected at day 15 in CD14^+^ cells.

Most pathway enrichment was detected at day 7 post-infection, and, similarly to B-cells, genes related to viral infection sensing and initiation of an immune response against the infection were upregulated in CD14^+^ cells. Additionally, downregulated genes involved in focal adhesion and cytoskeleton regulation were enriched in CD14^+^ cells.

##### 3.2.2.3 CD4^+^ cells

At day 3, upregulated DEGs were enriched in pathways involved in virus sensing (infectious disease: RNA virus and infectious disease: viral), and PRR signaling (NOD-like receptor, C-type lectin receptor and RIG-I- like receptor signaling) in CD4^+^ cells (**Figure 4**). Downregulated DEGs at day 3 were related to ribosome pathways. At day 7, an increase in the number of upregulated genes related to viral sensing and PRR signaling (C-type lectin receptor, NOD-like receptor and toll-like receptor signaling) was detected. DEGs involved in antigen processing and presentation, cytokine signaling (chemokine, NF-kappa B and TNF signaling), B and Th17-cell receptor signaling, and T-cell exhaustion (PD-L1 expression and PD-1 checkpoint) pathways were also upregulated in CD4^+^ cells at day 7. Downregulated DEGs at day 7 were involved in RNA degradation and ribosome pathways. No enrichment in pathways was detected at day 15 in CD4^+^ cells.

In CD4^+^ cells, upregulated DEGs were enriched in pathways related to viral sensing and initiation of immune response. However, in contrast with B and CD14^+^ cells, CD4^+^ cells also expressed upregulated genes involved in T-cell exhaustion pathway.

##### 3.2.2.4 CD8^+^ cells

Upregulated DEGs in CD8^+^ cells at day 3 were also involved in virus sensing (infectious disease: RNA virus and infectious disease: viral) and PRR signaling (RIG-I-like receptor signaling) (**Figure 4**). At day 7, upregulated DEGs participated in virus sensing, antigen processing (proteasome) and ribosome biogenesis pathways. Pathway enrichment downregulated genes were only detected at day 7 and were involved in PRR signaling (NOD-like receptor), T-cell receptor signaling, MAPK signaling, chemokine signaling, RNA degradation, cellular senescence, autophagy, leukocyte transendothelial transport, regulation of actin cytoskeleton, focal adhesion and long-term potentiation. CD8^+^ cells were the only population that showed upregulated gene expression enrichment in pathways at day 15. These genes were involved in T-cell differentiation, cytokine signaling (TNF signaling), lysosome, and T-cell exhaustion (PD-L1 expression and PD-1 checkpoint).

CD8^+^ upregulated genes were mainly enriched in virus sensing and PRR signaling pathways. In contrast to the other cell populations, CD8^+^ cells were the only population that showed downregulated gene expression in pathways involved in pathogen infection sensing and initiation of an immune response at day 7. Other genes related to pathways predicted to be upregulated during an immune response, such as T cell receptor signaling, transendothelial transport, and immune synapse (long term potentiation), were also downregulated at day 7. Additionally, CD8^+^ cells were the only population that had enriched upregulated genes in pathways at day 15. These were involved in the initiation of an immune response against the infection but also in T-cell exhaustion. ORA-KEGG analysis thus indicates that CD8^+^ T cell response to BTV is following a different kinetic to other immune cells, which is in line with the contraction in the periphery of these cells at day 7 followed by their expansion at day 15 detected by flow cytometry analysis (**Figure 2A**).

#### 3.2.3 Interferon-related genes are upregulated in B, CD14^+^, CD4^+^ and CD8^+^ cells

**Figure 5:**
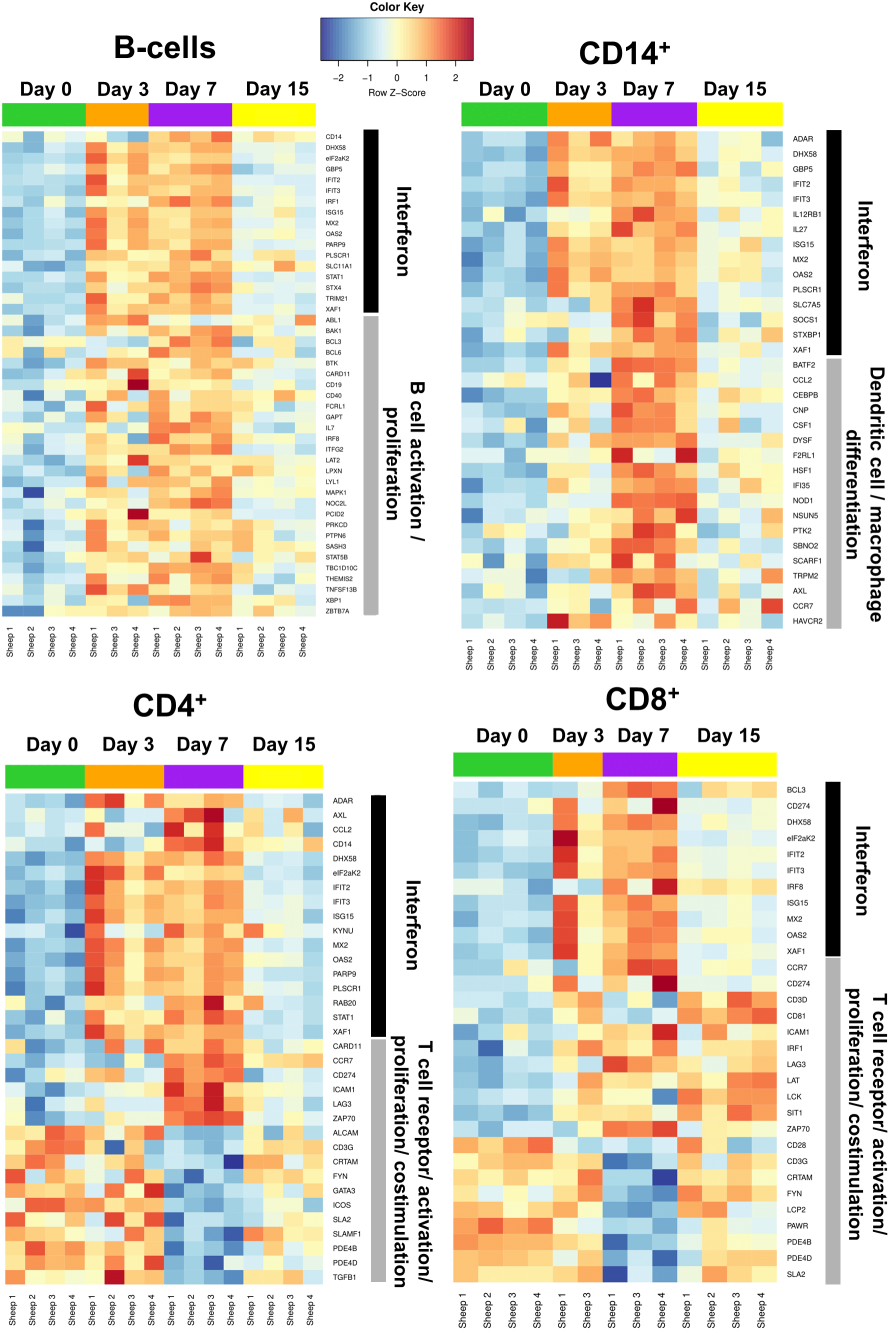
Heatmaps of selected genes associated with the immune response in B cell, CD14^+^, CD4^+^, and CD8^+^ cells. Heatmaps were generated based on the GO term “Interferon” for all 4 populations (black bar). Heatmaps were also generated using the GO terms: “B cell activation/proliferation” for sorted B cells; “Dendritic cell/macrophage differentiation” for sorted CD14^+^ cells; and “T cell receptor/activation/proliferation/costimulation” for sorted CD4^+^ and CD8^+^ cells (grey bar). Only samples with RIN>7.0 were processed for RNAseq (59 out of 64 possible samples).

Heat maps were plotted to study DEG expression in B, CD14^+^, CD4^+^ and CD8^+^ cells on selected gene ontology (GO) terms. Gene expression for these GO terms at days 3, 7 and 15 post-infection was compared to day 0 (prior to infection) (**Figure 5**).

Since the IFN response is essential for the establishment of antiviral immunity (50), the GO term “interferon” was selected in all four cell populations as it can encompass pathogen sensing and the initiation of immune responses against viral infection. The four cell types analyzed in this study showed increased expression of DEGs related to the GO term “interferon” at days 3 and 7, before their expression started to normalize by day 15 to reach levels similar to day 0 (**Figure 5**). It thus appears that B, CD14^+^, CD4^+^, and CD8^+^ cells are capable of sensing the infection and, as a consequence, trigger the transcription of IFN-related genes.

We also represented DEG heat maps based on GO terms related to the biological functions/activation of each cell type. B cells showed the highest expression of DEGs associated to the GO terms “B cell activation/proliferation” at days 3 and 7, which then decreased at day 15 to reach expression levels similar to those of day 0. We detected at day 3pi an upregulation in the expression of CD19, the B cell receptor co-receptor, as well as B-cell activating factor (BAFF/*TNFSF13B*), a potent B cell activator, which indicates that B cells could be activated early in the infection. Expression of CD14^+^ cells DEGs associated to the GO term “dendritic cell/macrophage differentiation” started to increase at day 3 and reached a maximum at day 7 before they started to decrease at day 15. Migratory chemokine *CCL2* and macrophage growth factor *CSF1* genes are upregulated at day 7pi, which indicates that an active response is taking place in monocytes. This is in line with the observation that the CD14^+^CD16^+^ monocyte population increased at day 7pi (**Figure 2D**). These data indicate that B and CD14^+^ cell activation is likely taking place in response to the infection.

#### 3.2.4 Immune checkpoint genes are upregulated in T cells at day 7pi

CD4^+^ DEGs associated to the GO term “T-cell receptor/activation/proliferation/costimulation” also reached maximum expression levels at day 7 before decreasing by day 15 (**Figure 5**). Interestingly, genes involved in T cell activation and in T cell inhibition are simultaneously differentially expressed at day 7pi. Expression of *CD3G*, *ICOS*, *ALCAM* and *SLAMF1*, which are important for T cell activation (51–53), is downregulated, while *ZAP70*, which is essential for TCR signal transduction (54), is upregulated. It is worth noting that immune checkpoint genes *CD274* and *LAG3* (55, 56) are overexpressed at this timepoint in CD4^+^ cells, which concurs with the ORA-KEGG pathway enrichment (**Figure 4**). It thus appears that a complex response to BTV is taking place in CD4^+^ T cells at day 7pi, although PD1-PD-L1 pathway enrichment and DEG expression is indicating that CD4^+^ T cell activity could be limited by the infection.

Similarly to CD4^+^ cells, CD8^+^ DEGs associated to the GO term “T-cell receptor/activation/proliferation/costimulation” showed maximum expression at day 7 (**Figure 5**). However, in contrast to CD4^+^ cells, DEG expression associated to these GO terms persisted until day 15 in CD8^+^ cells. At day 7pi, genes involved in T cell activation and inhibition were also concomitantly differentially expressed in CD8^+^ cells. DEGs indicating positive activation of CD8^+^ cells, such as *ZAP70* or *LAT*, were overexpressed at day 7pi, which suggests that CD8^+^ T cells are responding to the infection. Nonetheless, immune checkpoint genes *CD274* and *LAG3* (55, 56) and negative regulator of TCR signaling *SIT1* (57) were overexpressed at day 7pi. The TCR component *CD3G*, the costimulatory molecule *CD28*, and the component of the TCR signalosome *LCP2* (58) were also downregulated at this timepoint. The expression of the adhesion molecule *CRTAM* that promotes cytotoxicity in NK cells and IFN-γ production in CD8^+^ T cells (59) was also downregulated at day 7pi. DEG expression thus concurs with the pathway enrichment analysis in CD8^+^ cells that pointed at T cell receptor signaling downregulation. This also correlates with the decreased percentage of CD8^+^ T cells detected by flow cytometry at day 7pi (**Figure 2A**). By day 15, most DEGs associated to the GO terms “T-cell receptor/activation/proliferation/costimulation” in CD8^+^ cells were involved in the positive regulation of T cell activity, and importantly, no downregulated genes related to T cell activation were detected (**Figure 5**). This correlated with the expansion of the CD8^+^ T cell compartment in the periphery (**Figure 2A**). Transcriptomic and flow cytometry analysis is therefore indicating that BTV may be affecting T cell responses at day 7pi but that the host is capable of responding to the infection by day 15pi.

### 3.3 BTV infection compromises T cell responses at day 7pi

**Figure 6:**
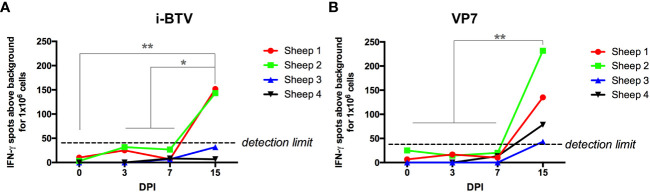
T cell responses to BTV antigens become detectable by day 15 post-infection. PBMCs isolated at day 0 (pre-infection), and day 3, 7 and 15 post-infection were stimulated with **(A)** BEI-inactivated BTV-8 (i-BTV) or **(B)** recombinant VP7 protein for 48h and IFN-γ production assessed in ELISpot assays. Data are presented as average IFN-γ production for 1x10^6^ PBMCs above background (unstimulated cells) for each sheep. *p<0.05; **p<0.01; One-way ANOVA with Fisher’s LSD post-test.

Given that immune checkpoint pathways were upregulated in T cells during the course of the infection, we next assessed the functionality of these cells. We first measured the T cell response to BTV in IFN-γ ELISpot assays (**Figure 6**). PBMCs from the four BTV-infected sheep collected at day 0 (before infection) and at days 3, 7 and 15 post infection were stimulated with inactivated BTV or a recombinant VP7 protein and IFN-γ production measured. T cell responses to inactivated BTV or recombinant VP7 became detectable in PBMCs at day 15 post-infection. This increase in IFN-γ production coincides with the CD8^+^ cell expansion detected by flow cytometry, as well as the upregulation of the TNF signaling pathways at day 15 in this cell population (**Figures 2A**, **4**). Infected sheep are therefore capable of mounting a T cell response to the infection by day 15pi.

**Figure 7:**
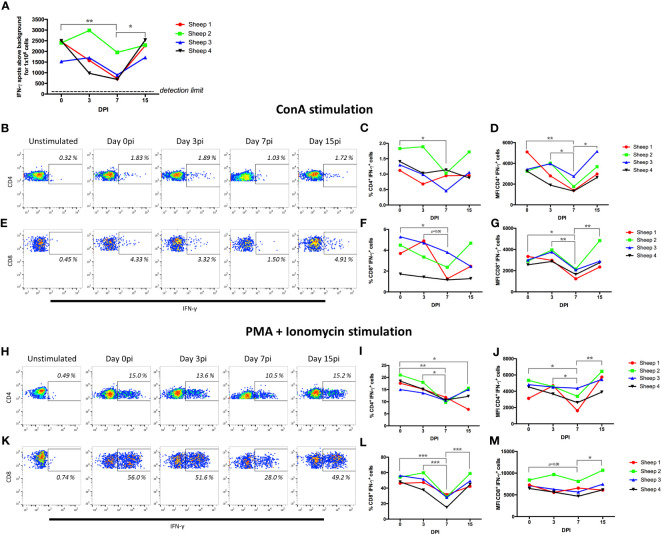
BTV-8 infection impairs CD4^+^ and CD8^+^ T-cell responses. **(A)** PBMCs isolated at day 0, 3, 7 and 15 post-infection (pi) (DPI) were stimulated with ConA and IFN-γ production assessed in ELISpot assays. PBMCs were stimulated with **(B-G)** ConA for 4 hours and IFN-γ production assessed in **(B-D)** CD4^+^ T cells and **(E-G)** CD8^+^ T cells in flow cytometry intracellular cytokine staining assays. Representative **(B)** IFN-γ/CD4 and **(E)** IFN-γ/CD8 dot-plots in ConA-stimulated PBMCs. Percentage and geometric mean fluorescence intensity (MFI) of IFN-γ^+^ cells that respond to ConA within **(C, D)** CD4^+^ or **(F, G)** CD8^+^ T cell gate at day 0, 3, 7 and 15 pi are shown. **(H-M)** PBMCs were stimulated with PMA + ionomycin for 4 hours and IFN-γ production assessed in **(H-J)** CD4^+^ T cells and (**K-M)** CD8^+^ T cells by flow cytometry. Representative **(H)** IFN-γ/CD4 and **(K)** IFN-γ/CD8 dot-plots in PMA + ionomycin stimulated PBMCs. Percentage and geometric mean fluorescence intensity (MFI) of IFN-γ^+^ cells that respond to PMA + ionomycin within the **(I, J)** CD4^+^ or **(L, M)** CD8^+^ T cell gate at day 0, 3, 7 and 15 pi for are shown. *p<0.05; ** p<0.01; ***p<0.001. One-way ANOVA with Fisher’s LSD post-test.

Because our transcriptomic analysis indicated that at day 7pi the PD1/PD-L1 checkpoint pathway was upregulated in CD4^+^ cells while the T cell receptor signaling pathway was downregulated in CD8^+^ cells, we also assessed T cell functionality using mitogen and activators. PBMCs from the four BTV-infected sheep were stimulated with the mitogen concanavalin A (ConA), and T-cell activation assessed using IFN-γ ELISpot assays at day 0 (previous to infection) and at days 3, 7, and 15pi (**Figure 7A**). ConA stimulation led to IFN-γ production in all animals at all timepoints, however a significant decrease in the number of IFN-γ-producing cells was detected at day 7pi in all sheep when compared to the baseline (day 0). At day 15pi, IFN-γ production was restored to similar levels to day 0. These data indicate that T cell responses were impaired in the periphery at day 7pi by the infection.

We next assessed which T cell population was affected by this reduction in activation. We carried out intracellular cytokine staining (ICS) and flow cytometry analysis for IFN-γ in CD4^+^ and CD8^+^ T cells that were stimulated with ConA (**Figures 7B–G**), or with a mixture of the activators phorbol 12-myristate 13-acetate (PMA) and ionomycin that induces a more potent T-cell activation than ConA (**Figures 7H–M**). The percentage of CD4^+^ T cells and CD8^+^ T cells producing IFN-γ in response to ConA stimulation was significantly decreased at day 7pi when compared to day 0 (**Figures 7C, F**). Importantly, when we measured the fluorescence intensity of the IFN-γ^+^ events, we found that this was also significantly reduced at day 7pi when compared to day 0, and to day 3 and 15pi for both T cell populations (**Figures 7D, G**). This is therefore indicating that the infection is not only reducing the number of CD4^+^ and CD8^+^ T cells responding to the mitogen ConA, but that it is also limiting the production of IFN-γ in the responding cells.

To confirm this finding, we stimulated the cells with PMA and ionomycin, a potent cocktail that induces strong cytokine production in T cells, and assessed IFN-γ production in CD4^+^ and CD8^+^ T cells by ICS and flow cytometry (**Figures 7H–M**). As predicted, PMA and ionomycin treatment resulted in a higher frequency of IFN-γ producing cells in both CD4^+^ and CD8^+^ T cells than ConA stimulation. These cells also produced higher amounts of IFN-γ as indicated by the higher MFI of IFN-γ^+^ events. Remarkably, we detected a significant decrease in the frequency of CD4^+^ and CD8^+^ T cells that produced IFN-γ in response to PMA and ionomycin stimulation at day 7pi when compared to day 0 or day 3pi (**Figures 7I, L**). Similarly to the data obtained with ConA stimulation, MFI of IFN-γ^+^ events was significantly decreased in CD4^+^ T cells at day 7pi when compared to day 0, 3pi and 15pi (**Figure 7J**); while in CD8^+^ T cells MFI tended to decrease at day 7pi when compared to day 0 and was significantly reduced when compared to day 15pi (**Figure 7M**). We also performed proliferation assays on T cells using the mitogens ConA and the superantigen SEB (Staphylococcus Enterotoxin B), which mediates activation through the TCR (60), using PBMC obtained at day 0 and day 7pi (**Supplementary Figure 7**). The proliferative capacity of CD4^+^ and CD8^+^ T cells was reduced at day 7pi with both mitogens when compared to day 0, further confirming that BTV impairs T cell functions in the periphery at day 7pi. Taken together, these data indicate that BTV infection is producing a severe impairment in CD4^+^ and CD8^+^ T cell function at day 7pi. This functional data confirms the transcriptomic analysis that detected at day 7pi the upregulation of T-cell exhaustion pathway and the downregulation of T cell receptor signaling in CD4^+^ and CD8^+^ cells respectively.

### 3.4 BTV infection increases PD-L1 expression in T cells and monocytes

**Figure 8:**
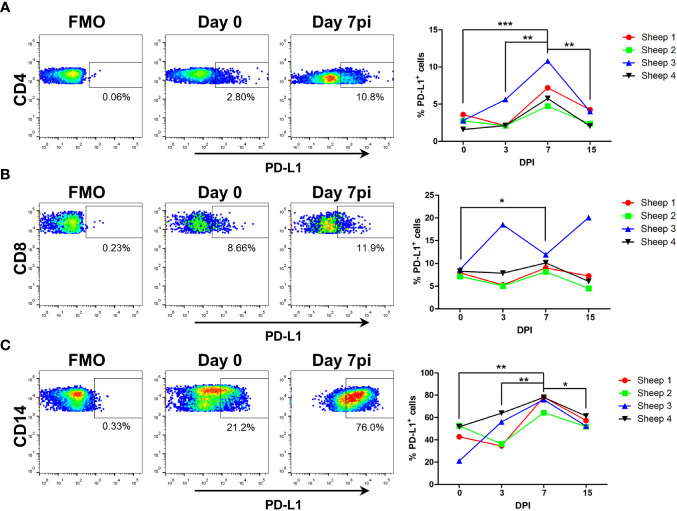
PD-L1 expression increases on CD4^+^, CD8^+^, and CD14^+^ cells at day 7 post-BTV infection. Sheep PBMC obtained prior to infection (D0) and at day 3, 7 and 15 post-BTV infection (DPI) were stained with anti-CD4, -CD8, -CD14 and –PD-L1 antibodies and percentage of PD-L1^+^ cells within each PBMC subpopulation determined by flow cytometry. **(A-C)** Representative dot-plots at day 0, 3, 7, and 15pi for PD-L1 expression in **(A)** CD4^+^, **(B)** CD8^+^, and **(C)** CD14^+^ cells. Fluorescence minus one (FMO) and isotype controls were used to set PD-L1 gates. (**Supplementary Figure 2**). Percentage of PD-L1^+^ cells within **(A)** CD4^+^, **(B)** CD8^+^, and **(C)** CD14^+^ cells at day 0, 3, 7, and 15pi are plotted. *p<0.05; ** p<0.01; ***p<0.001. One-way ANOVA with Fisher’s LSD post-test.

PD-L1 expression in T cells and antigen-presenting cells is known to limit T cell activation (61). Since transcriptomic analysis showed that PD-L1 gene (CD274) is overexpressed in CD4^+^ T cells, CD8^+^ T cells and monocytes at day 7pi (increased log fold-change 1.80; 2.60; and 1.83 respectively) (**Supplementary File 1**), we assessed the surface expression of the protein on these cells by flow cytometry (**Figure 8**). The percentage of PD-L1 expressing cells increased at day 7pi in all three PBMCs subpopulations when compared to day 0, which confirmed the transcriptional data. This percentage normalized by day 15pi, which indicated that the increased number of PD-L1^+^ cells is a transient phenomenon triggered by the infection process. Increased PD-L1 expression in T cell subsets at day 7pi could therefore explain the impaired T cell activity. Interestingly, PD-L1 expression also augmented in circulating monocytes at day 7pi. PD-L1 expression on antigen presenting cells impairs T cell activation, and thus PD-L1 expression on monocytes could contribute to further suppress T cell activation at this timepoint. Taken together these data indicate that BTV infection promotes PD-L1 overexpression in immune cells to hinder T cell response development.

## 4 Discussion

In the present work, we characterized the transcriptome of B cells, CD4^+^ and CD8^+^ T cells, and monocytes in response to *in vivo* BTV infections in the natural host. To perform this analysis, we experimentally infected 4 sheep with BTV-8. All animals developed severe BT disease, with high fever, apathy, and lack of appetite that peaked at day 7pi. Viremia was still readily detectable by RT-qPCR at the end of the experiment (day 17pi), which is characteristic of the prolonged presence of virus in circulation during BTV infections (20). B cell and monocyte transcriptome indicated that these cells are involved in viral recognition at early timepoints of the infection. We observed a severe inhibition of polyclonal CD4^+^ and CD8^+^ T cell responses at day 7pi, which coincided with the upregulation of the PD1/PD-L1 axis in CD4^+^ T cells, and the downregulation of TCR signaling in CD8^+^ T cells. Functional analysis of T cells responses showed that both CD4^+^ and CD8^+^ T cells activation was impaired at day 7pi, a hallmark of immune checkpoint activation pathways in T cells. Our data also show that BTV induces PD-L1 expression in T cells and monocytes, a process that could mediate T cell response attenuation.

Previous transcriptomic analysis of *in vitro* BTV infection of ovine and caprine PBMCs pointed at the activation of genes related to immunity, which belonged, among others, to PRR and cytokine/chemokine signaling pathways (34). In the present work, we present the transcriptomic analysis of 4 PBMC subpopulations after *in vivo* BTV infection in sheep. We found that IFN-related genes DHX58 (LGBP2), IFIT2, IFIT3, ISG15, MX2, OAS2, and XAF1 were upregulated at day 3 and 7pi in all 4 PBMC subpopulations. Singh et al. also reported that IFIT2 and IFIT3 are upregulated in sheep and goat PBMCs infected *in vitro* with BTV-16 (34). IFIT expression is unconventional among IFN-stimulated genes. IFITs are typically endogenously expressed at low levels since they can act as sensor for the infection by recognizing viral RNA motifs (62). Indeed, IFIT2 can recognize dsRNA while IFIT3 modulates IFIT2-driven apoptosis (63), thus it is plausible that IFIT2 could participate in BTV RNA recognition. A recent study also showed that IFIT1 is important in restricting BTV replication in bovine and ovine primary cells (64). IFIT expression is also induced upon IFN signaling. IFITs can inhibit viral protein translation and promote antiviral signal transduction. Our data therefore confirms that IFIT could play an important role in the host innate immune response to BTV. Further work analyzing IFIT contribution to BTV immunity is nonetheless required to address the precise role of these IFN-stimulated genes in BTV infection. The upregulation of IFN-related genes in B, CD14^+^, CD4^+^, and CD8^+^ cells is indicating that the host is detecting the presence of the viral infection and consequently activating the IFN response. This is a predictable outcome since BTV has been described as a good inducer of IFN, and infection leads to IFN production in cattle and sheep (65–67). Differences in the IFN response kinetics between cattle and sheep could actually be responsible for host susceptibility to disease (64).

Our comprehensive transcriptomic analysis also indicated that B cells and monocytes are involved in the sensing of the infection from day 3pi. Pathway enrichment analysis indicated that cell activation occurs for B cells and monocytes, at least in the periphery, following BTV infection. Upregulation of genes involved in B cell activation, such as *CD19* or *TNFSF13B*, occurred by day 3pi, although this was not maintained at day 7pi. We have previously described that BTV can target follicular DC to delay antibody responses (27). Although our transcriptomic analysis did not detect a significant impairment of B cell activity in the periphery, we found that *STAT5B* was upregulated at day 7pi. STAT5B has recently been identified as a repressor of the immunoglobulin class switch and the transcriptional program that leads to plasma cell differentiation (68). Thus, it could be speculated that transcriptional mechanisms could be promoted by the infection to delay antibody production in spite of the apparent activation of B cells. Alternatively, follicular DC disruption by the infection could be sufficient to impair B cell activation locally and delay the antibody response. Further characterization of the B cell response to BTV will be required to better elucidate the intricate regulation of antibody production in this infection.

Our flow cytometry analysis of monocytes also showed that the CD14^+^CD16^+^ monocyte subpopulation expanded by day 7pi. Expansion of this monocyte subset has also been reported during PPRV infection in sheep (69). This monocyte population may therefore play a role in the ovine immune response to viral infections. Full characterization of this CD14^+^ CD16^+^ monocyte population would require the introduction of other surface markers, such as CD172a as previously done in cattle (49), which would allow for a clearer differentiation between classical, activated and intermediate/non-classical monocytes. Although monocyte subset functions have not been fully characterized in sheep (48), their bovine counterparts appear to share similar activity to the human equivalent monocyte population (70, 71). Recent studies have further confirmed this shared phenotype of monocytes in cattle and humans at the transcriptomic level (49, 72). Broadly speaking in human and cattle, classical monocyte subsets can drive a pro-inflammatory response while non-classical monocyte subsets are thought to be anti-inflammatory and involved in vascular repair. Along with the upregulation of IFN-related genes in monocytes, we found that proinflammatory cytokine genes *IL23A*, *TNF* and *IL6* were upregulated at day 7pi, which coincided with the increased presence of CD14^+^CD16^+^ cells in the monocyte subset. This could indicate that an inflammatory response is promoted by monocytes at day 7pi and that activation of classical monocytes is occurring. Nonetheless, we concomitantly detected a consistent upregulation of the *IL27* gene throughout the infection. IL-27 is typically associated with the induction of the regulatory cytokine IL-10 and the promotion of T regulatory cell activity, although it can also be critical for CD8^+^ T cell activation (reviewed in (73)). CD14^+^ cell transcriptomic analysis also revealed that the genes expressing the immune checkpoint proteins TIM-3 and PD-L1 (*HAVCR2* and *CD274*) are upregulated at day 7pi. We confirmed the increased expression of PD-L1 protein on monocyte surface by flow cytometry at this timepoint. Expression of these immune checkpoint molecules could represent a counterpoint to immune activation and the need to dampen inflammation to prevent immunopathology. These data indicate that this CD14^+^CD16^+^ monocyte subset could also include intermediate/non-classical monocytes, which could be important to repair the vascular damage produced by BTV infection. Interestingly, PD-L1 expression in non-classical monocytes has been linked to immunoregulatory functions (74), and transcriptomic studies in cattle have found that this monocyte subset expresses higher levels of PD-L1 and IL-27 (49). In future work it will be interesting to determine the specific PD-L1 expression on CD14^+^CD16^+^ monocytes, as this would help characterize this monocyte subset. Talker et al. have proposed a model of continuous differentiation for monocytes in cattle (49). They suggest that the CD14^+^CD16^+^ population could represent an heterogeneous intermediate monocyte subset that is transitioning from a classical inflammatory profile towards a non-classical anti-inflammatory profile. Our bulk transcriptomic analysis of CD14^+^ cells during viral infection also indicates that the enrichment in circulating CD14^+^CD16^+^ coincided with the upregulation of both inflammatory and anti-inflammatory gene sets in monocytes, which could fit this model. Overall, it appears that a complex response to the infection is taking place in monocytes. On the one hand, upregulation of PRR and TNF signaling pathways indicate that monocytes participate in the recognition of the infection and the initiation of the immune response, but on the other hand, expression of potent inhibitors of immunity such as IL-27, TIM-3 (75, 76), or PD-L1 (74) could also limit the stimulatory activity of these cells and promote an immunoregulatory environment. The balance between these signals will probably modulate the response of surrounding cells. It should be noted that our current transcriptomic analysis was based on CD14^+^-sorted cells, and as a result, this will encompass several monocyte subsets. Transcriptomic profiling of the different monocyte populations in sheep could shed some light on their function, and consequently help interpret the transcriptomic response of monocytes in infection.

We started detecting anti-BTV cellular immunity by day 15pi. This is in line with other reports that show that humoral and cellular anti-BTV immunity can be detected from day 9 post-infection (24), and that CD8^+^ T and B cell expansion occurs by day 10-12 at the lymph node proximal to the infection site (25). In spite of the mounting immune response, BTV is still capable of replication in the host as shown by the presence of virus in peripheral blood throughout our experimental infection. Several mechanisms of immune escape have been described for BTV. We have shown that BTV can target follicular DC to delay antibody responses (27). BTV is also known to suppress IFN responses via some of its non-structural and structural proteins (77–81). Although these mechanisms contribute to BTV immune evasion, they are unlikely to fully account for BTV capacity to prolong its circulation in the host.

Transcriptomic analysis of CD4^+^ and CD8^+^ T cells indicated that T cell responses could be disturbed at day 7pi. This coincided with the decreased number of CD8^+^ T cells in the periphery at this timepoint. Functional studies confirmed the transcriptomic data, and we found that CD4^+^ and CD8^+^ T cell activation was severely impaired at day 7 pi when compared to day 0. The limited capacity of T cell mitogens and activators to trigger a response in day 7pi cells is therefore indicative that the infection has imprinted in the periphery a program in T cells that impairs activation. These impaired T cell responses correlated with the activation of the PD-1/PD-L1 axis according to our transcriptomic data. The PD-1/PD-L1 axis is an immune checkpoint that controls T cell exhaustion, and its inhibition has proved useful for antitumor immunotherapy. Activation of the PD-1/PD-L1 axis appears to be a natural process in acute viral infections that controls excessive T cell activation, prevents immunopathogenicity, and maintains peripheral tolerance (82). The activation of the PD-1/PD-L1 axis in T cells is associated with an increase in the activation threshold required to trigger TCR signaling (83), and thus this could account for the impaired T cell phenotype observed upon BTV infection. In β-coronavirus infections, such as murine hepatitis virus and SARS-Cov2, the PD1/PD-L1 axis is involved in T cell dysfunction (84). Indeed, viruses that produce chronic infections can exploit the PD1/PD-L1 axis, among other immune checkpoints, to exhaust virus-specific T cells (82).

Although the canonical PD-1/PD-L1 exhaustion pathway involves engagement of PD-1 on T cells, our transcriptomic analysis showed it was actually PD-L1 (*CD274*) expression that was upregulated both in CD4^+^ and CD8^+^ T cells at day 7pi. Flow cytometry analysis confirmed an increase in the subsets of PD-L1 expressing-CD4^+^ and -CD8^+^ T cells at day 7pi. Upregulation of PD-L1 in CD4^+^ T cells has previously been reported in *in vitro* HIV infections (85). PD-L1 expression is upregulated in immune cells in response to pro-inflammatory cytokines or type I IFN (82). PD-L1 can be expressed in activated T cells and studies in PD-L1-deficient mice have shown that its presence on T cells limits activation (61). Indeed, in cancer PD-L1 activation on T cells weakens antitumor immunity (86), while PD-L1 signaling on human memory CD4^+^ T cells induces a regulatory phenotype (87). PD-L1 engagement on T cells promotes self-tolerance and suppresses the activity of neighboring effector T cells and macrophages (86). PD-L1^+^ T cells can impair T cell activity by interacting with PD-1 on effector cells. PD-L1 also reduces Th1 polarization and promotes Th17 differentiation in CD4^+^ T cells (86). Our transcriptomic pathway analysis showed that Th17 signaling was upregulated in CD4^+^ T cells at day 7pi, and thus it could be speculated that PD-L1 engagement could contribute to this finding. *LAG3* upregulation is taking place concomitantly to PD-L1 expression in both T cell subsets at day 7pi. *LAG3* also belongs to the immune checkpoint family, and probably works synergistically with PD-1 to promote T cell exhaustion (88). *LAG3* upregulation on CD4^+^ and CD8^+^ T could therefore also contribute to the immunosuppressed phenotype of T cells at this timepoint. It is noteworthy that *IL10* expression is also upregulated in CD4^+^ T cells at day 7pi, and this could also participate in the immunosuppression since this cytokine is key in immune modulation (89). Taken together, our data indicate that BTV infection is triggering the activation of several immune regulatory processes.

Immune checkpoint proteins are typically triggered to resolve inflammation and limit tissue damage. Indeed, blockade of PD-L1 and depletion of Tregs in viral infections improves viral clearance, but leads to the development of immunopathology (90). Thus, BTV could be taking advantage of T cell regulatory loops that are in place to prevent tissue damage to expand its window of replication in the host. The activation of an inflammatory response resulting from the vascular damage produced by BTV infection in endothelial cells would need to be countered by potent inhibitors of inflammation such as IL-27. IL-27 promotes PD-1/PD-L1 and IL-10 expression (73), and we found that it was upregulated in the monocyte population throughout the infection. In this context, the virus could take advantage of the regulatory pathways that dampen inflammation to promote T cell immunosuppression at the peak of viral replication, for instance by inducing immune checkpoint protein expression such as PD-L1 and LAG-3 on T cells and by promoting IL-27 and PD-L1 expression in monocytes. Our data indicate that BTV is probably profiting from regulatory mechanisms to limit T cell responses and implicate PD-L1 expression on T cells and monocytes as a possible mechanism to suppress the host immune response.

Ultimately, our data show that the host mounts T cell responses capable of recognizing BTV by day 15pi. This coincided with the expansion of CD8^+^ T cells in the periphery, and the upregulation of *IFNG* in CD8^+^ cells in our transcriptomic analysis. Transcriptomic analysis of CD8^+^ cells also showed that no gene involved in TCR signaling were downregulated at day 15pi, suggesting that T cell activity has been normalized at this timepoint. Thus, in spite of the potent inhibitory effects of the infection at day 7pi on T cells, this immunosuppression appears to be transient and does not prevent the development of anti-BTV T cells. It remains unclear whether this immunosuppressive window has affected the quality of the T cell repertoire that responds to BTV. Multiple BTV infections reduce the spectrum of T cell epitopes recognized by the host (21), and thus BTV can affect its recognition by T cells. A more in depth analysis of the T cell phenotypes (effector/memory) generated upon BTV infections will need to be carried out to better evaluate the T cell activity during and after the infection.

Taken together, these data highlight the capacity of BTV to manipulate regulatory pathways in T cells, probably to limit its recognition and further its replication since cellular immunity is involved in disease protection (29–32). Further understanding of the interactions between the host immune system and BTV could help better comprehend the mechanisms that allow BTV to prolong its circulation in spite of the mounting immune response. Ultimately, these studies can provide veterinary medicine with better tools to combat the disease.

## Data availability statement

The datasets presented in this study can be found in online repositories. The names of the repository/repositories and accession number(s) can be found below: GSE233994 (GEO).

## Ethics statement

The animal study was approved by Committee on the Ethics of Animal Experiments of the Spanish Instituto Nacional de Investigación y Tecnología Agraria y Alimentaria (INIA) and the National Animal Welfare Committee. The study was conducted in accordance with the local legislation and institutional requirements.

## Author contributions

AL-L: Investigation, Writing – original draft, Writing – review & editing, Data curation, Formal Analysis, Methodology. JR: Formal Analysis, Investigation, Methodology, Writing – original draft, Writing – review & editing, Conceptualization, Supervision. IG-G: Investigation, Writing – review & editing, Data curation, Software. DR-M: Investigation, Writing - review & editing, Methodology. EM: Investigation, Writing - review & editing, Methodology. VM: Funding acquisition, Supervision, Writing – review & editing. NS: Funding acquisition, Supervision, Conceptualization, Investigation, Project administration, Writing – original draft, Writing – review & editing.
