# Supplementary figures and images for: Comprehensive immune profiling reveals that Orbivirus infection activates immune checkpoints during acute T cell immunosuppression
Source: Front Immunol. 2023 Oct 18;14:1255803. doi: 10.3389/fimmu.2023.1255803 (PMC10619675; doi:10.3389/fimmu.2023.1255803)

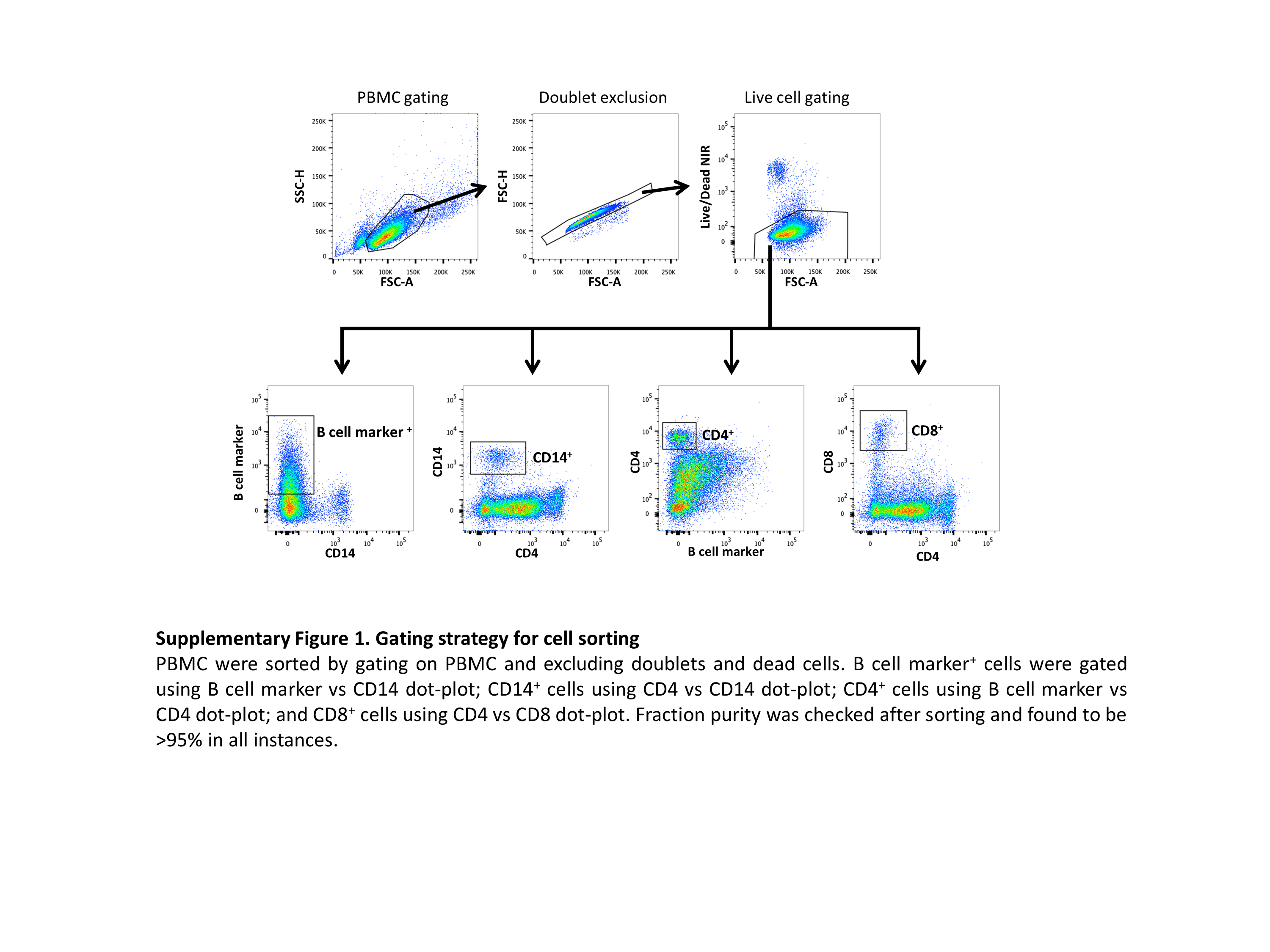

Supplement: Supplementary file 2 [file Image_1.tif]

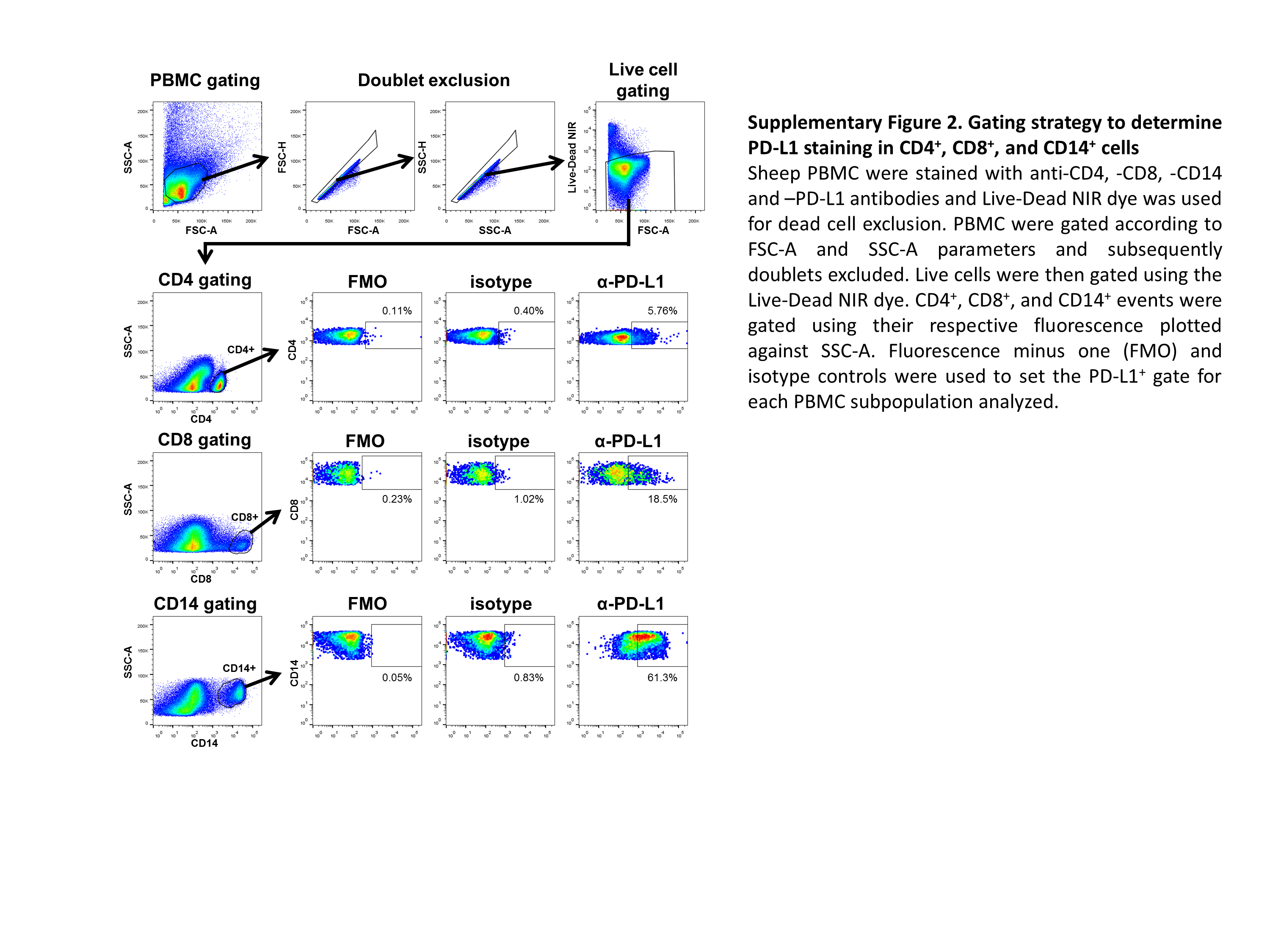

Supplement: Supplementary file 3 [file Image_2.tif]

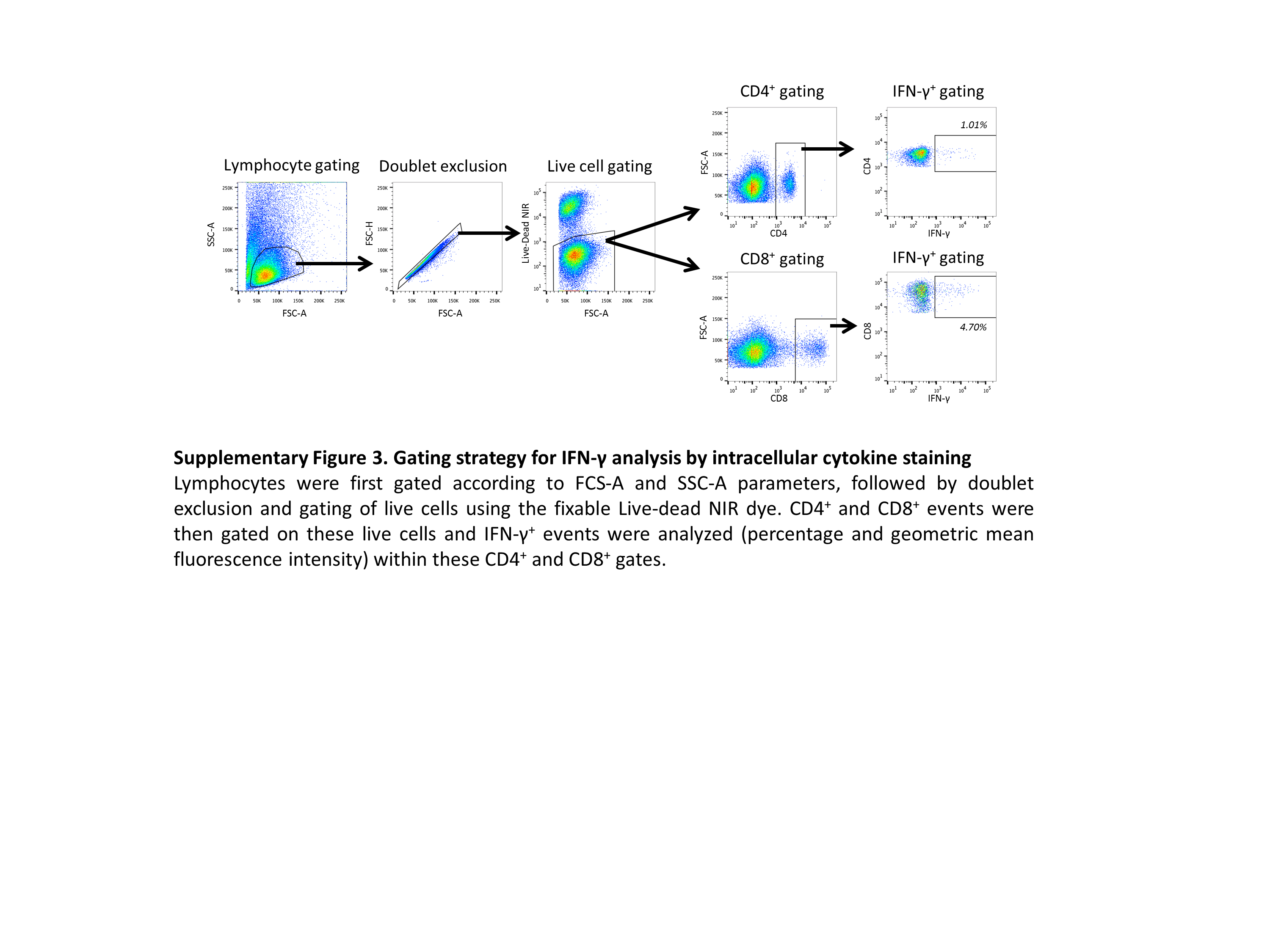

Supplement: Supplementary file 4 [file Image_3.tif]

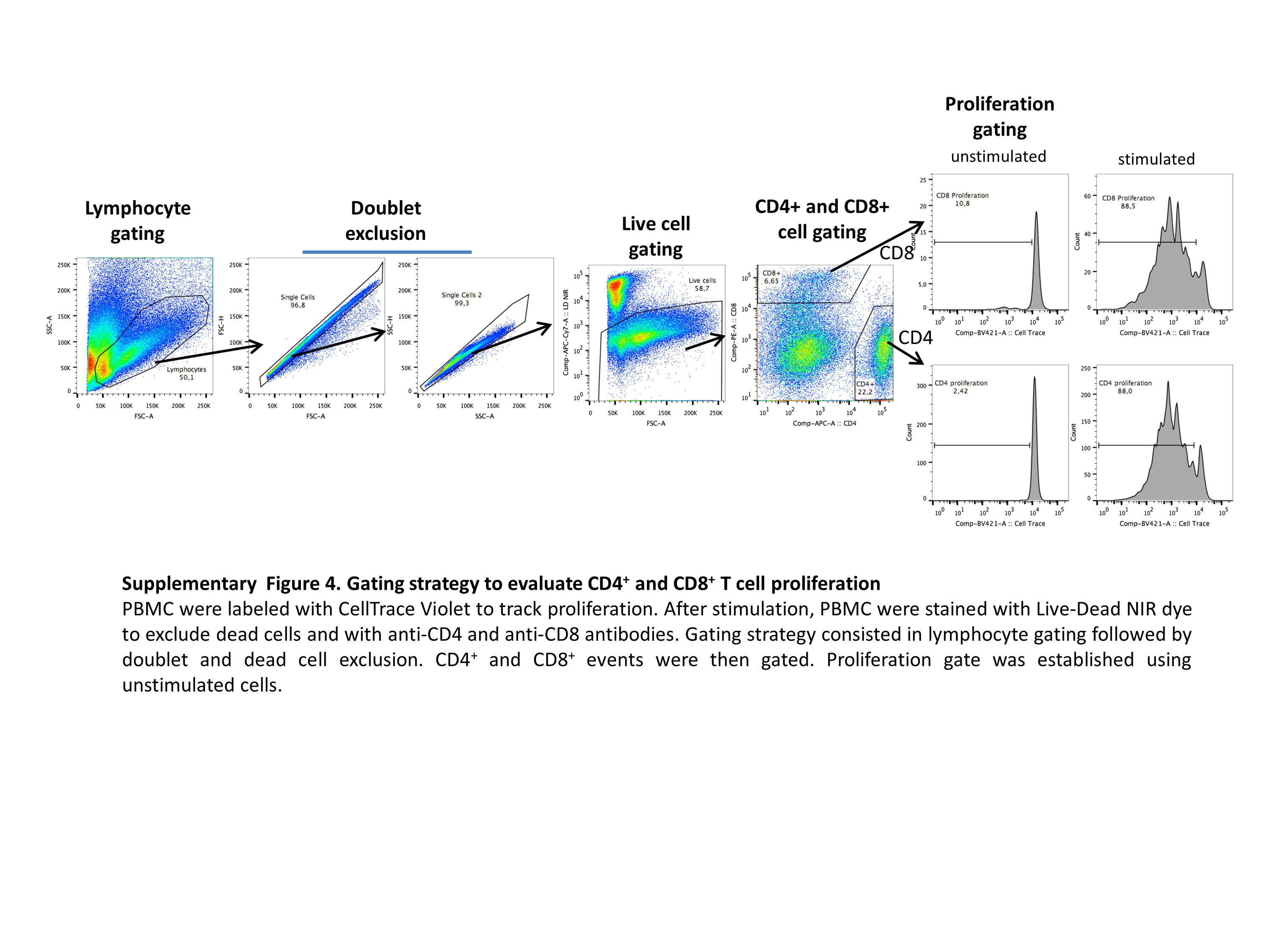

Supplement: Supplementary file 5 [file Image_4.tif]

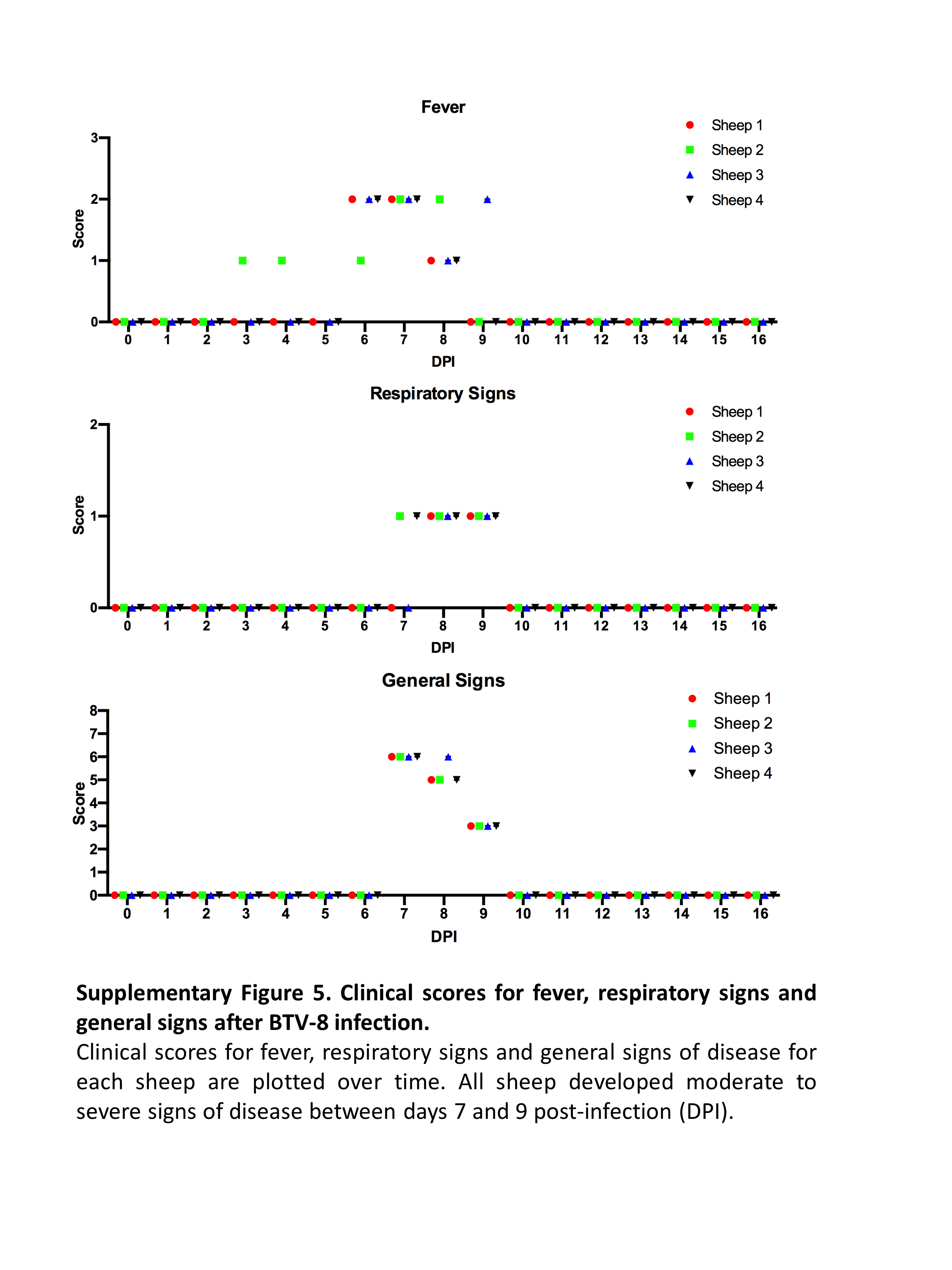

Supplement: Supplementary file 6 [file Image_5.tif]

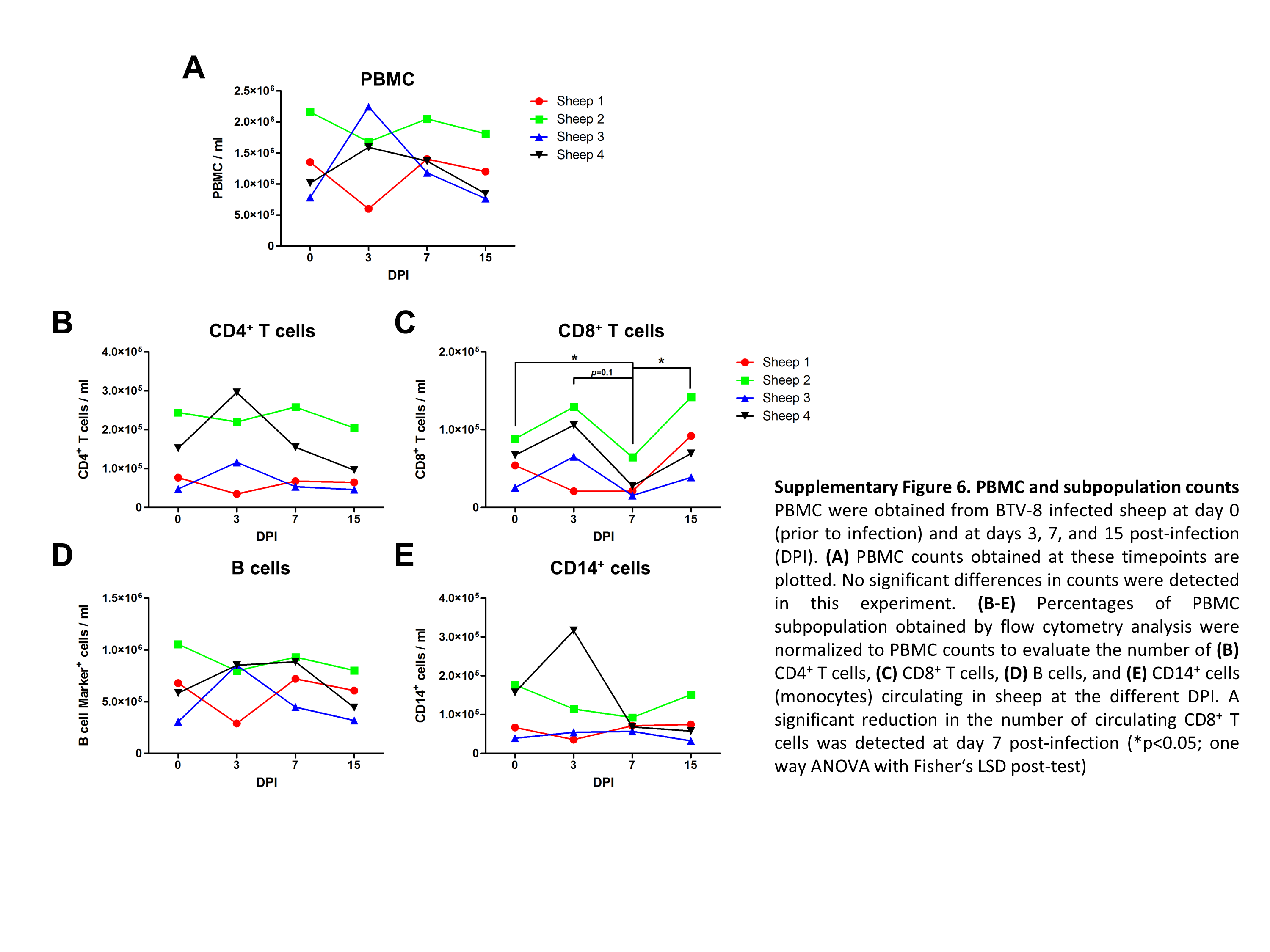

Supplement: Supplementary file 7 [file Image_6.tif]

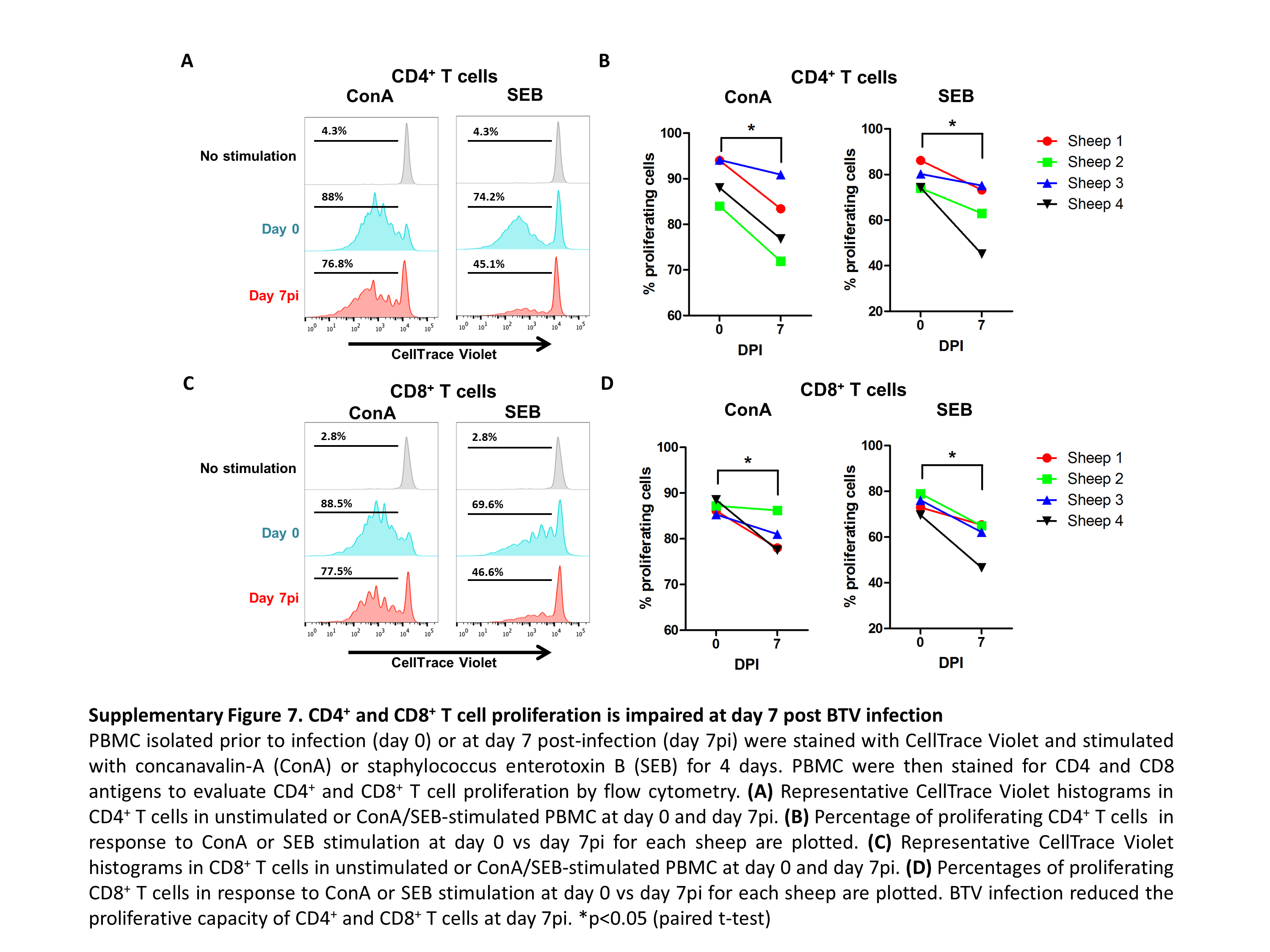

Supplement: Supplementary file 8 [file Image_7.tif]
