# Supplementary material for: Comprehensive immune profiling reveals that Orbivirus infection activates immune checkpoints during acute T cell immunosuppression
Source: Front Immunol. 2023 Oct 18;14:1255803. doi: 10.3389/fimmu.2023.1255803 (PMC10619675; doi:10.3389/fimmu.2023.1255803)
Supplement: Supplementary file 9 [file Table_1.pdf]

Supplementary Table 1. B cell marker, CD4, CD8, and CD14 sorted fraction counts

Day 0							
**Sheep #1**		**Sheep #2**		**Sheep #3**		**Sheep #4**	
B	3x10^{6}	B	1.7x10^{6}	B	2.3x10^{6}	B	2.5x10^{6}
CD4	1.5x10^{6}	CD4	2.4x10^{6}	CD4	2.3x10^{6}	CD4	3x10^{6}
CD8	5.6x10^{5}	CD8	6.3x10^{5}	CD8	7.72x10^{5}	CD8	5.96x10^{5}
CD14	8.35x10^{5}	CD14	9.2x10^{5}	CD14	8.77x10^{5}	CD14	1.7x10^{6}

Day 3pi							
**Sheep #1**		**Sheep #2**		**Sheep #3**		**Sheep #4**	
B	2.4x10^{6}	B	4.95x10^{6}	B	2.19x10^{6}	B	3.4x10^{6}
CD4	1.48x10^{6}	CD4	4.68x10^{6}	CD4	1.6x10^{6}	CD4	3.17x10^{6}
CD8	6.69x10^{5}	CD8	3.48x10^{6}	CD8	6.67x10^{5}	CD8	8.6x10^{5}
CD14	8.09x10^{5}	CD14	1.2x10^{6}	CD14	6.4x10^{5}	CD14	2.69x10^{6}

Day 7pi							
**Sheep #1**		**Sheep #2**		**Sheep #3**		**Sheep #4**	
B	4.25x10^{6}	B	3.4x10^{6}	B	3.02X10^{6}	B	1.64x10^{6}
CD4	1.26x10^{6}	CD4	1.89x10^{6}	CD4	1.05x10^{6}	CD4	7.39x10^{5}
CD8	2.87x10^{5}	CD8	5.23x10^{5}	CD8	3.61x10^{5}	CD8	1.33x10^{5}
CD14	4.16x10^{5}	CD14	3.29x10^{5}	CD14	4.14x10^{5}	CD14	2.16x10^{5}

Day 15pi							
**Sheep #1**		**Sheep #2**		**Sheep #3**		**Sheep #4**	
B	5x10^{6}	B	4.96x10^{6}	B	3.65x10^{6}	B	4.78x10^{6}
CD4	2.073x10^{6}	CD4	3.45x10^{6}	CD4	2.78x10^{6}	CD4	2.9x10^{6}
CD8	2.363x10^{6}	CD8	3.26x10^{6}	CD8	1.54x10^{6}	CD8	2.22x10^{6}
CD14	2.23x10^{6}	CD14	1.63x10^{6}	CD14	7.37x10^{5}	CD14	6.25x10^{5}
